# Lightweight real-time detectors of apple-leaf diseases operating on embedded devices

**DOI:** 10.1371/journal.pone.0352500

**Published:** 2026-07-09

**Authors:** Yujie Qin, Qingyang Liu, Hongjiu Liu, Haiyang Zhang, Zhanlin Ji, Ivan Ganchev

**Affiliations:** 1 College of Mathematics and Computer Science, Zhejiang A&F University, Hangzhou, China; 2 School of Advanced Technology, Xi’an Jiaotong-Liverpool University, Suzhou, China; 3 TRC/ECE, University of Limerick, Plassey, National Technological Park, Limerick, Ireland; 4 Faculty of Mathematics and Informatics, University of Plovdiv “Paisii Hilendarski”, Plovdiv, Bulgaria; 5 Institute of Mathematics and Informatics – Bulgarian Academy of Sciences, Sofia, Bulgaria; University of the West of Scotland, UNITED KINGDOM OF GREAT BRITAIN AND NORTHERN IRELAND

## Abstract

Agricultural leaf disease detection is crucial for early intervention and yield protection in precision agriculture. Among representative economic crops, such as apples, leaf lesions are typically small and appear in complex backgrounds, making accurate detection performed on resource-constrained embedded devices challenging. To address this, we propose a lightweight small-object detection models, namely the dynamic Differential Compensation Lightweight-YOLO (DCL-YOLO) model and its pruned version (DCL-YOLO-P), based on YOLO11n. A novel Dual-Aspect Feature Complementary Mapping (DAFCM) module type is embedded in their backbone to recover lost semantic and spatial information, while the original YOLO11n’s neck is replaced by an Efficient Enhanced Cross-Scale Feature Fusion (EE-CSFF) module, which incorporates Gated Differential Convolutional Fusion (GDCF) modules to strengthen cross-scale information flow and small-object representation. Experimental results obtained on the ALDSOD dataset show that, compared with the YOLO11n baseline, DCL-YOLO improves recall from 81.9% to 84.6%, mAP_50_ from 86.8% to 88.4%, and mAP_50:95_ from 47.0% to 47.8%, while also reducing the parameter count from 2.58 M to 1.91 M and Giga Floating-Point Operations (GFLOPs) from 6.3 to 5.5. After applying Layer-Adaptive Magnitude-based Pruning (LAMP), the parameter count and GFLOPs are further reduced to 0.75 M and 2.7, respectively, with mAP_50_ and mAP_50:95_ still exceeding the baseline by 1.2 and 0.5 percentage points, respectively. When deployed on an embedded device, the pruned model achieved 15.2 FPS and 139 msec per image, confirming its applicability in real-time scenarios. Furthermore, cross-domain validation, performed on the Global Wheat Head Detection (GWHD) dataset, indicates the stable generalization capabilities of the proposed models across environmental domain shifts. The DCL-YOLO’s source code is publicly available at: https://github.com/q123-code/dcl-yolo.

## 1. Introduction

Apples are a major global economic crop, playing a crucial role in fruit production, agricultural development, and global nutrition [1,2]. Under natural orchard conditions, high temperature and humidity combined with pathogen spread make apple trees highly susceptible to foliar diseases such as Alternaria leaf spot, rust, gray spot, and frogeye leaf spot [3]. Early lesions are small (2–5 mm), morphologically diverse, and often obscured by complex leaf textures or variable lighting, leading to high miss rates in manual inspection and traditional image-processing methods [4,5]. With the increasing adoption of intelligent orchard management, accurate and efficient disease monitoring has become essential, providing the foundation for automated orchard tasks such as precise spraying and yield estimation. At the same time, edge-based detection systems typically operate on mobile or embedded platforms with limited computation and memory. Therefore, accurately detecting small lesions while maintaining lightweight neural-network architecture and real-time performance remains a major challenge, and existing approaches still face the following three key obstacles:

1. **Inherent mismatch between deep semantic and shallow spatial features:** One major difficulty in early-stage lesion detection arises from the inherent mismatch between deep semantic and shallow spatial features as backbone networks deepen. Feature Pyramid Networks (FPNs) [6] address this issue by enabling top-down fusion of high-level semantics with low-level spatial details, thereby enhancing multi-scale representation and small-object localization. However, for apple-leaf small lesions, shallow-layer features often contain the most critical fine-grained edge and texture cues. Conventional FPNs, which rely on fixed backbone structures, may fail to fully preserve these features, reducing the ability to distinguish tiny spots from the surrounding leaf surface.
2. **Rigidity of feature fusion mechanisms:** Pyramid-based detection frameworks typically use static concatenation (Concat) and fixed convolution for feature fusion, assuming fixed receptive fields and uniform weighting. To mitigate this issue, Dong et al. [7] proposed CTAFFNet, a Convolutional Neural Network (CNN)–Transformer dual branch with Squeeze-and-Excitation (SE) and Multilayer Perceptron (MLP) for adaptive local and global feature fusion, alleviating rigid convolutional fusion. Yet, when applied to apple leaves, these methods still struggle to maintain the saliency of extremely small or weakly responsive lesions, as subtle color and texture differences can be diluted across layers or spatial locations. Moreover, the lack of explicit gradient paths hinders the model’s ability to learn and reinforce relevant features, ultimately degrading the detection accuracy and robustness. This limitation is particularly critical for early-stage disease spot detection in apple leaves, representing a key bottleneck requiring urgent resolution.
3. **Structural loss of spatial detail during downsampling:** Repeated downsampling and feature map compression can weaken the representation of small lesions, thereby limiting effective cross-scale interaction. Preserving fine-grained spatial information while ensuring robust fusion of semantic and spatial cues across layers thus remains difficult. Although several approaches have attempted to alleviate this problem by enhancing feature alignment or improving multi-scale fusion [8,9], their effectiveness is still constrained by incomplete retention of shallow-layer details, limited flexibility in cross-layer information exchange, and insufficient responsiveness to extremely small or weakly expressed targets.

To address the challenges discussed above, this study proposes novel lightweight small-object detection models, namely DCL-YOLO and its pruned version (DCL-YOLO-P), based on YOLO11n. The latter has been successfully deployed on an embedded device, achieving real-time and efficient edge inference. The main contributions of this article can be summarized as follows:

1. To alleviate the feature loss of small lesions caused by the mismatch between deep semantic information and shallow spatial details, a newly designed Dual-Aspect Feature Complementary Mapping (DAFCM) module is proposed for utilization by the proposed models. Unlike generic attention mechanisms that often treat all features equally and tend to suppress minute pathological textures, it employs a tailored dual-path strategy to adaptively compensate for the information loss that occurs during the interaction between deep semantic layers and shallow spatial layers. By specifically balancing these two information streams, this module design distinguishes itself from prior attention blocks by effectively enhancing feature alignment for highly camouflaged small objects – a capability that is often suboptimal in standard attention blocks.
2. A novel Gated Differential Convolutional Fusion (GDCF) module type is proposed for use by the proposed models to address the potential information degradation caused by static fusion strategies. In contrast to conventional context aggregators that rely on computationally expensive or static operations, GDCF employs a differential-aware gating mechanism, which allows the network to dynamically filter redundant orchard background noise and enhance feature responses of small objects and weakly activated regions. This transitions the mechanism from static feature addition to dynamic noise-aware filtering, improving detection accuracy while maintaining an efficient design and low computational overhead.
3. To address the challenge of reducing model complexity while enhancing cross-scale feature interaction, a newly designed Efficient Enhanced Cross-Scale Feature Fusion (EE-CSFF) module is proposed for utilization by the proposed models. Departing from closely related prior designs that heavily rely on dense convolutions and incur substantial memory access costs (MAC), EE-CSFF employs explicit channel alignment and multi-path fusion. This edge-efficient pathway not only enhances information flow and preserves shallow features but also reduces model parameters and computational costs, effectively improving optimizing hardware friendliness for resource-constrained edge deployments.
4. The Layer-Adaptive Magnitude-based Pruning (LAMP) method is applied to the proposed DCL-YOLO-P model, achieving substantial reductions in model’s parameter count, computational cost, and size under controlled accuracy loss. Combined with UINT8 quantization, the pruned DCL-YOLO-P model (with a size of 1.24 MB) was successfully deployed on an embedded device, where it achieved a real-time edge inference at 15.2 FPS, demonstrating practicality for resource constrained devices.

The remainder of the article is organized as follows. The Related Work section reviews small-object detection models and deep learning applications in apple-leaf disease detection. The Materials and Methods section provides a detailed description of the proposed model’s architecture and pruning strategy. The Experiments section introduces the datasets used and presents the comprehensive experimental results along with corresponding discussions. The Lightweight K230 Embedded Deployment section describes the model implementation on a hardware platform with evaluation of the deployment performance. Finally, the Conclusion section summarizes the article and proposes directions for future research.

## 2. Related work

### 2.1. Small-object detection

Object detection models are generally divided into two-stage and one-stage types. One-stage models, such as the YOLO series [10,11], have been widely adopted for small-object detection. Despite their popularity and recent improvements in accuracy and speed, these models still face significant challenges. They often struggle with low coverage of small objects, sparse feature information, and limited image resolution. To address these issues, Hou et al. [12] proposed Multi-stage Feature Enhancement Lightweight-YOLO (MFEL-YOLO), a model designed specifically for small-object detection. It incorporates a Hybrid C2F (HE-C2F) module for feature extraction, which employs deformable convolution to adaptively capture multi-scale features and utilizes a grouped multi-branch structure to enhance small-object feature representation in Unmanned Aerial Vehicles (UAV) images. Furthermore, Peng et al. [13] introduced (mixed scene-oriented small-object Detection Transformer) MI-DETR, which uses Fast Fourier Transform (FFT), channel shuffling, and orthogonal attention to enhance global context modeling. It also includes a Multi-scale Feature Fusion (MSFF) module to better preserve small-object features. In addition, the Focaler WIoU loss is adopted to optimize bounding box regression and improve localization accuracy. Zhang et al. [14] proposed DsP-YOLO, an anchor-free YOLOv8-based model for industrial small-defect detection. It incorporates a Detail-sensitive PAN (DsPAN) module that focuses on enhancing features of small objects.

Furthermore, recent advances in lightweight small-object detection have increasingly focused on aerial and agricultural scenarios. For instance, Zhou et al. [15] proposed the LMFF-MFFE model, which utilizes a multiscale dense feature fusion network and multi-receptive field feature enhancement to balance parameter count and detection accuracy in resource-constrained UAV imagery. Similarly, in the context of crop disease detection, Yu et al. [16] developed BGM-YOLO by integrating a median-enhanced spatial and channel attention block (MECS) and a bitemporal fusion module to capture small-scale leaf spot features amidst complex natural backgrounds. Although the aforementioned models have made notable progress in small-object feature extraction and fusion, a significant limitation still remains – most recent lightweight models primarily focus on general or industrial scenarios. When applied to agricultural environments, they often struggle to adequately handle the high degree of camouflage of subtle lesions against complex orchard backgrounds. Standard lightweight designs tend to rely heavily on downsampling, leading to significant spatial information loss for minute targets. To address these challenges, it is important to select a detection framework that is both mature and efficient. The YOLO series has consistently set the benchmark in real-time object detection. In particular, YOLO11 [17], with its flexibility and ease of use, represents a widely adopted and well-established framework, which makes it an excellent foundation for further improvement. Building upon this foundation, this study proposes the DCL-YOLO model, based on YOLO11n, which enhances feature matching capability, response representation strength, and multi-scale information interaction efficiency w.r.t. small objects, while maintaining lightweight architecture.

### 2.2. Object detection for apple-leaf disease

With the advancement of research, object detection has become the dominant approach for the identification of apple-leaf diseases, delivering notable performance improvements. For instance, Yan et al. [18] proposed FSM-YOLO, an improved CNN for apple-leaf disease detection in unstructured environments. It integrates an Adaptive Feature Enhancement Module (AFEM) for adaptive feature enhancement, a Spatial Context-aware Attention (SCAA) module for spatial context-aware attention, and a Multi-kernel Mixed Convolution (MKMC) for multi-scale feature extraction, achieving improvements of 2.7% in mAP_50_, 2.0% in precision, and 4.0% in recall over the baseline (YOLOv8s). Despite progress in lesion detection, existing models typically involve a large number of parameters, limiting their deployment in mobile devices and real-time agricultural scenarios. To address this limitation, Xu et al. [19] developed a lightweight framework, ALAD-YOLO, which combines an efficient backbone network with an attention mechanism to improve feature representation while reducing model complexity.

To further bridge the gap between theoretical models and hardware deployment, recent studies have increasingly focused on deployment-aware methods in precision agriculture. For example, Wen et al. [20] improved the YOLO11 architecture for sugarcane stem node detection by combining an attentional scale sequence fusion mechanism with a lightweight shared detection head, and subsequently applied channel pruning to successfully compress the model to a mere 1.3 MB for field deployment. Indeed, pruning has been widely adopted as an effective post-training compression technique in object detection, where connections or neurons with weights below a given threshold are removed to yield a sparser network. However, the effectiveness of pruning depends on the degree of redundancy in the network. To address this, Lee et al. [21] proposed the LAMP scoring method, which incorporates a model-level $l_{\text{2}}$ distortion induced by pruning to determine layer-wise sparsity. Overall, lightweight architectures and pruning techniques have jointly facilitated the practical deployment of models on mobile platforms in this field.

## 3. Materials and methods

**Fig 1:**
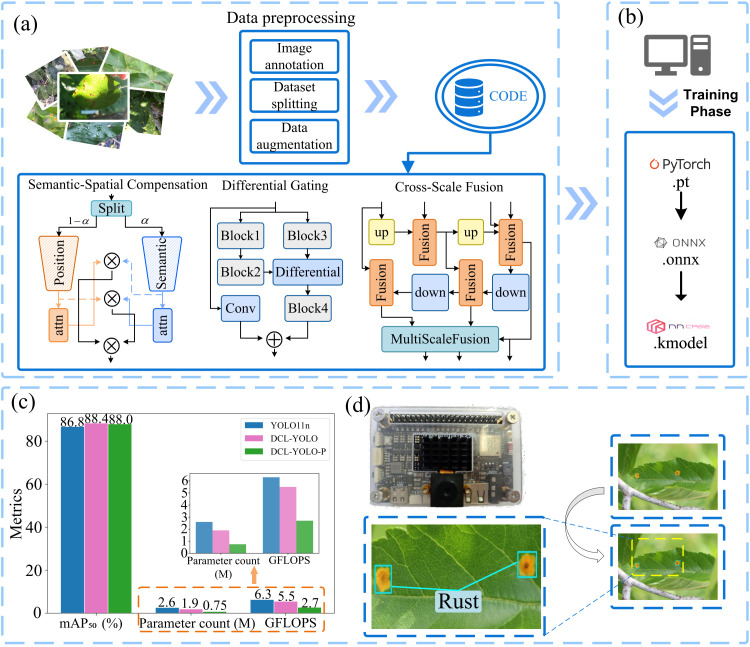
The overall framework of the proposed apple-leaf disease detection methodology. (a) Data pipeline and DCL-YOLO architecture (featuring Semantic-Spatial Compensation, Differential Gating, and Cross-Scale Fusion). (b) Model training on PC and *nncase* toolchain conversion to*.kmodel*. (c) Performance comparison to the baseline (YOLO11n). (d) K230 hardware deployment, showing real-time inference with cyan boxes localizing “Rust” lesions. Overall, the proposed models (especially DCL-YOLO-P) achieve superior detection performance with a significantly lighter footprint for efficient edge deployment..

This section begins with an introduction to the first proposed model (DCL-YOLO), built upon YOLO11n for detecting apple-leaf lesions in complex orchard environments. Subsequently, a detailed account is provided of its architectural improvements designed to address the limitations of the baseline model, along with their implementation specifics. Finally, the pruned version (DCL-YOLO-P) of it is described. The overall framework of the proposed apple-leaf disease detection methodology is illustrated in Fig 1.

### 3.1. Proposed models

**Fig 2:**
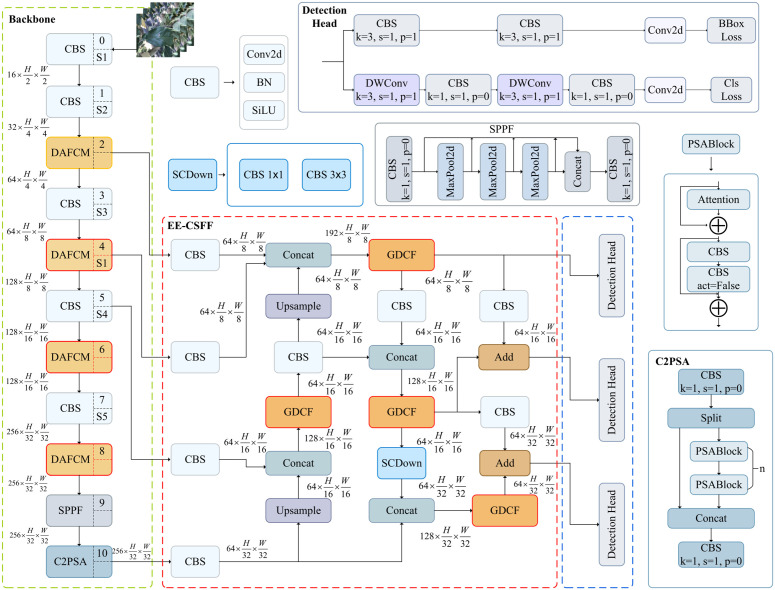
The overall network architecture of the proposed DCL-YOLO model. The backbone (*left*) extracts hierarchical features via Conv-BatchNorm-SiLU (CBS), Dual-Aspect Feature Complementary Mapping (DAFCM), Spatial Pyramid Pooling-Fast (SPPF), and C2PSA (H and W denote spatial dimensions). The Efficient Enhanced Cross-Scale Feature Fusion (EE-CSFF) neck (*center*) aggregates multi-scale representations through GDCF and SCDown. The head (*right*) generates predictions using a depthwise convolution (DWConv). Peripheral boxes detail sub-modules, where k, s, and p represent kernel size, stride, and padding. Overall, this design integrates multi-scale features to enhance small lesion detection with minimal computational overhead.

The network architecture of the proposed DCL-YOLO model, shown in Fig 2, integrates three novel key components that operate cohesively across the backbone and neck. Within the backbone, DAFCM modules adaptively recover deep semantic cues and shallow spatial details through a dual-path compensation strategy, producing more informative feature maps for downstream processing. The original YOLO11n’s neck is replaced with the newly designed EE-CSFF module, which integrates novel GDCF modules to facilitate cross-scale feature propagation and enhance the representation of small and weakly activated targets, resulting in a comprehensive feature map which is better suited for small and occluded objects prior to detection.

### 3.2. DAFCM module

In CNNs, mismatches between deep semantic features and shallow spatial features often lead to information loss during feature fusion, which adversely affects the detection of small lesions. The conventional Cross Stage Partial with kernel size 2 (C3k2) module [17] alleviates this issue by concatenating features along the channel dimension followed by convolution, but it does not adequately balance high-level semantic information and low-level spatial details. Deep features provide strong category discrimination, but lose precise localization due to downsampling and channel compression. In contrast, shallow features retain rich texture and edge information, but lack semantic discriminability.

**Fig 3:**
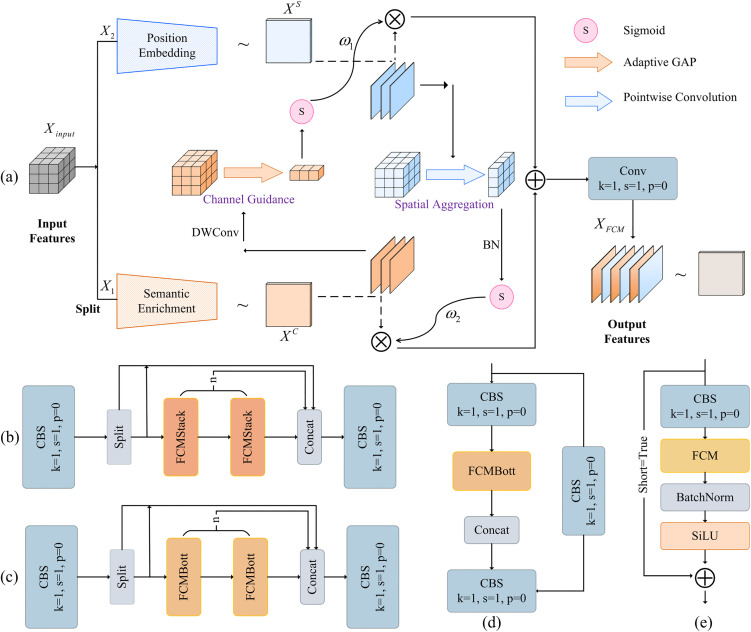
The detailed architecture of the newly designed DAFCM module, utilized by the proposed models. (a) The core Feature Complementary Mapping (FCM) unit processes input features via a dual-path design (Position Embedding and Semantic Enrichment), which allows to compensate for missing spatial-semantic information, thus improving downstream detection performance; (b) & (c) DAFCM configurations with C3K=True and C3K=False, respectively. (d) An FCMStack; (e) An FCMBott block embedding an FCM unit. Pink circles (S) denote a sigmoid activation function, the orange arrow represents an adaptive global average pooling (GAP), and the blue arrow indicates a pointwise convolution.

To address this limitation, we designed a novel DAFCM module, which integrates a Feature Complementary Mapping (FCM) unit [22] into the original C3k2 structure, as shown in Fig 3. FCM (shown in Fig 3a) employs a dual-path strategy, splitting input features along the channel dimension into spatial embedding and semantic enrichment streams to capture spatial details and semantic information, respectively. The former stream generates a spatial attention map to suppress irrelevant background, while the latter stream produces a channel attention map to emphasize key semantic channels. The two attention maps are cross-weighted to highlight discriminative information along both spatial and channel dimensions, and the fused features further compensate for missing information.

Specifically, in the FCM unit, the input feature map $X_{input}\in R^{C\times H\times W}$ is split along the channel dimension into two portions according to α and 1−α (c.f., Fig 3a). Subsequently, the first αC channels are assigned to the semantic enrichment branch as input $X_{\text{1}}$, while the remaining (1−α)C channels are fed into the position encoding branch as input $X_{\text{2}}$, where 0≤α≤1. This channel splitting process can be expressed as follows:


$$\begin{array}{c} \left(X_{\text{1}},X_{\text{2}})=\text{Split}(\text{X}\right) \end{array}$$ (1)

where $X_{\text{1}}\in R^{\alpha C\times H\times W}$, $X_{\text{2}}\in R^{(\text{1}-\alpha )C\times H\times W}$.

$X_{\text{1}}$ is passed through a branch composed of standard 3×3 convolutions, which extracts richer high-order semantic features from each channel denoted $X^{C}$. Meanwhile, $X_{\text{2}}$ is fed into a branch composed of pointwise convolutions, which capture relatively weak semantic information while preserving abundant shallow spatial location cues, denoted as $X^{S}$. This process is expressed as follows:


$$\begin{array}{c} \left(X^{C},X^{S})=\varphi (X_{\text{1}},X_{\text{2}}\right) \end{array}$$ (2)

where φ denotes the learned mapping between spatial and semantic information. The feature $X^{C}\in R^{C\times H\times W}$ carries rich channel-wise information, whereas $X^{S}\in R^{C\times H\times W}$ preserves more of the original spatial location details.

Although both features, $X^{C}$ and $X^{S}$, are effective, they lack complementary feature alignment, which may lead to misalignment in target representation. To address this issue, a complementary mapping is established between them as follows:


$$\begin{array}{c} \omega_{\text{1}}=\sigma (\text{GAP}(\text{DWCon}{\text{v}}_{\text{3}\times \text{3}}(X^{C}))) \end{array}$$ (3)

$$\begin{array}{c} \omega_{\text{2}}=\sigma (\text{BN}(\text{Con}{\text{v}}_{\text{1}\times \text{1}}(X^{S}))) \end{array}$$ (4)

where $\omega_{\text{1}}\in R^{C\times \text{1}\times \text{1}}$ and $\omega_{\text{2}}\in R^{\text{1}\times H\times W}$ denote the channel and spatial attention weights, respectively, GAP denotes a global average pooling, $\text{DWCon}{\text{v}}_{\text{3}\times \text{3}}\;$denotes a 3×3 depthwise convolution (applied independently to each channel), σ denotes a sigmoid activation function, BN denotes a batch normalization, and $\text{Con}{\text{v}}_{\text{1}\times \text{1}}\;$denotes an 1×1 convolution.

Specifically, the semantic feature $X^{C}$ is processed by a depthwise convolution, which performs independent convolutions within each channel to extract intra-channel responses. Subsequently, a lightweight channel attention is applied, which performs GAP to capture global information across channels, followed by a sigmoid layer to produce channel-wise importance weights $\omega_{\text{1}}$.

For spatial feature $X^{S}$, a spatial attention generation is applied by an 1×1 convolution, batch normalization, and a sigmoid function, generating spatial importance weights $\omega_{\text{2}}$.

**Algorithm 1** FCM unit operation.

1: **Input:** Feature map $X\in R^{C\times H\times W}$

2: **Output:** Feature map $X_{FCM}\in R^{C_{out}\times H\times W}$

3: **Split** X **into two parts:**

4: $X_{\text{1}}\in R^{\alpha C\times H\times W}$, $X_{\text{2}}\in R^{(\text{1}-\alpha )C\times H\times W}$, where 0≤α≤1

5: **Process semantic branch:**

6: $X^{C}\leftarrow$standard 3×3 convolutions on $X_{\text{1}}$

7: **Process spatial branch:**

8: $X^{S}\leftarrow$point-wise convolutions on $X_{\text{2}}$

9: **Generate attention weights:**

10: $\omega_{\text{1}}=\sigma (\text{GAP}(\text{DWCon}{\text{v}}_{\text{3}\times \text{3}}(X^{C})))$     ⊳ From semantics

11: $\omega_{\text{2}}=\sigma (\text{BN}(\text{Con}{\text{v}}_{\text{1}\times \text{1}}(X^{S})))$       ⊳ From spatial

12: **Apply attention mappings:**

13: ${\tilde{X}}^{S}\leftarrow \omega_{\text{1}}⊗X^{S}$          ⊳ Modulated by semantics

14: ${\tilde{X}}^{C}\leftarrow \omega_{\text{2}}⊗X^{C}$          ⊳ Modulated by spatial

15: **Fuse features (element-wise addition):**

16: $X_{\text{FCM}}\leftarrow \text{Con}{\text{v}}_{\text{1}\times \text{1}}({\tilde{X}}^{S}⊕{\tilde{X}}^{C})$       ⊳ Feature fusion

17: **return** $X_{\text{FCM}}$

After obtaining the channel weight $\omega_{\text{1}}$ and the spatial weight $\omega_{\text{2}}$, these are mapped to the features $X^{S}$ and $X^{C}$, respectively. Analytically, this cross-modulation explicitly maximizes the mutual information between spatial and semantic subspaces, while the sigmoid function strictly bounds the attention weights within [0, 1] to suppress background noise. Finally, rather than a standard concatenation, the two branches are fused via element-wise addition (⊕). According to gradient flow analysis, this additive residual pathway ensures independent gradient propagation (e.g., $\frac{\partial L}{\partial X^{S}}$ and $\frac{\partial L}{\partial X^{C}}$) during backpropagation, which effectively mitigates the vanishing gradient problem for small-lesion representations. This mapping and fusion process is formally specified as:


$$\begin{array}{c} X_{\text{FCM}}=\text{Con}{\text{v}}_{\text{1}\times \text{1}}((\omega_{\text{1}}⊗X^{S})⊕(\omega_{\text{2}}⊗X^{C})) \end{array}$$ (5)

where ⊗ denotes an element-wise multiplication with broadcasting semantics, and ⊕ denotes an element-wise addition. Algorithm 1 details the entire operation of the FCM unit.

### 3.3. GDCF module

**Fig 4:**
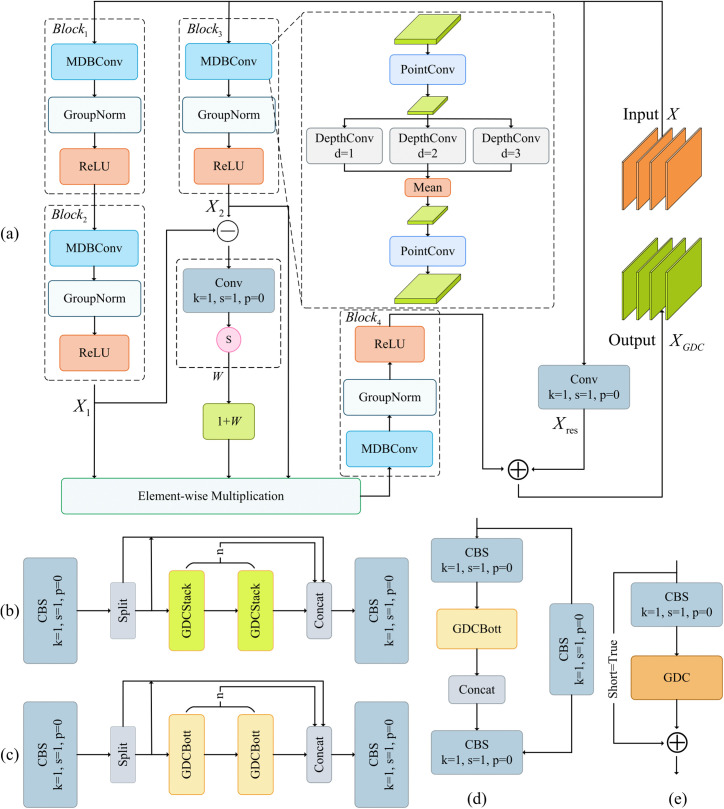
The detailed architecture of the newly designed GDCF module, utilized by the proposed models. (a) The core GDC unit featuring a differential-aware gating mechanism and Multi-Dilation Block Convolution (MDBConv) units, extracting multi-scale context via pointwise convolutions (PointConv) and depthwise convolutions (DepthConv) with varying dilation rates (*d*). (b) & (c) GDCF configurations with C3K=True and C3K=False, respectively. (d) A GDCStack. (e) A GDCBott block embedding the GDC unit, using Conv-BatchNorm-SiLU (CBS) for feature aggregation. Overall, this differential design adaptively amplifies responses for small objects and weakly activated regions, ensuring robust representations. Symbols ⊖, ⊕, and ⓢ denote an element-wise subtraction, an element-wise addition, and a sigmoid function, respectively.

To address information loss in weakly-responsive lesion regions, we designed a novel GDCF module, as illustrated in Fig 4. The original YOLO11’s neck C3k2 fusion nodes rely on standard convolution and conventional bottlenecks, lacking mechanisms to dynamically enhance small-object features. Inspired by the GBC module [23], we designed a more flexible Gated Differential Convolution (GDC) unit and integrated it with C3k2, resulting in the GDCF module. The GDC unit employs a differential-aware gating mechanism and multi-scale dilated convolutions to capture richer contextual information and improve detection of small objects with weak textures. Unlike the hard-multiplicative fusion in GBC, GDC adaptively modulates responses across spatial regions to avoid over-suppression.

In the GDC unit, the input feature map $X\in R^{C\times H\times W}$ is processed by a dual-branch architecture, which enables collaborative enhancement between shallow and deep features. Specifically, the deep branch (the left one in Fig 4a) consists of two consecutive Multi-Dilation Multi-Dilation Block Convolution (MDBConv) units, each followed by GroupNorm and activation functions. This branch captures contextual semantic information within a larger receptive field. The output of this branch is denoted as $X_{\text{1}}\in R^{C_{mid}\times H\times W}$. Meanwhile, the shallow branch (the right one in Fig 4a) utilizes a single MDBConv unit followed by GroupNorm and ReLU, compressing the input into a $C_{mid}$ dimensional space to retain local fine-grained details, with the output denoted as $X_{\text{2}}\in R^{C_{mid}\times H\times W}$. The MDBConv operation can be expressed as follows:


$$\begin{array}{c} \text{MDBConv}\left(𝐗\right)=\text{Con}{\text{v}}_{\text{1}\times \text{1}}\left(F_{\text{avg}}\right) \end{array}$$ (6)

$$\begin{array}{c} F_{\text{avg}}=\frac{\text{1}}{\text{3}}\sum_{d=\text{1}}^{\text{3}}\text{DWCon}{\text{v}}_{k,d}\left(\text{Con}{\text{v}}_{\text{1}\times \text{1}}\left(X\right)\right) \end{array}$$ (7)

where $\text{DWCon}{\text{v}}_{k,d}(\cdot )$ denotes a depthwise separable convolution with kernel size k and dilation rate d, and $F_{\text{avg}}$ denotes the element-wise average of the multi-scale convolution outputs.

Conceptually, this subtractive operation functions as a learnable high-pass filter, efficiently isolating fine-grained structural boundaries and edge discrepancies, which are critical for lesion localization. This structure efficiently integrates multi-scale spatial information while maintaining low computational complexity. To highlight the differential information between the deep branch and shallow branch, a difference map is computed as follows:


$$\begin{array}{c} D=X_{\text{1}}-X_{\text{2}} \end{array}$$ (8)

This difference map is then passed through a 1×1 convolution with a sigmoid activation to generate the spatial attention map $W\in [\text{0},\;\text{1}{]}^{C_{mid}\times H\times W}$, which is subsequently used to modulate the semantic features:


$$\begin{array}{c} {\tilde{X}}_{\text{1}}=X_{\text{1}}⊗\left(\text{1}+W\right) \end{array}$$ (9)

Analytically, the (1+W) term introduces identity mapping prior. By lower-bounding the modulation factor to 1, it prevents complete suppression of deep features, thereby maintaining the variance of the original semantic distribution and ensuring numerical stability during gradient updates. By adaptively modulating deep features, GDC mitigates information loss caused by low attention weights, while simultaneously emphasizing regions with the most significant discrepancies, thereby highlighting potential lesion signals more clearly.

The modulated deep features ${\tilde{X}}_{\text{1}}$ are then fused with shallow features by an element-wise multiplication, followed by an MDBConv for feature compression and refinement to align the output-channel dimensions. Meanwhile, to retain the original input information and improve the stability of the gradient propagation, the input X is projected through a 1×1 convolution to generate a residual branch $X_{\text{res}}\in R^{C_{out}\times H\times W}$. Finally, the module output is obtained by element-wise residual addition, yielding the feature $X_{\text{GDC}}\in R^{C_{out}\times H\times W}$.

This feature fusion and residual updating process can be formally expressed as follows:

**Algorithm 2** GDC unit operation.

1: **Input:** Feature map $X\in R^{C\times H\times W}$

2: **Output:** Feature map $X_{GDC}\in R^{C_{out}\times H\times W}$

3: **Definition:**

4: $Block_{i}(X)\leftarrow \text{ReLU}(\text{GN}(\text{MDBConv}(X)))$

5: $\text{MDBConv}(X)\leftarrow \text{Con}{\text{v}}_{\text{1}\times \text{1}}(\frac{\text{1}}{\text{3}}\sum_{d=\text{1}}^{\text{3}}\text{DWCon}{\text{v}}_{k,d}(\text{Con}{\text{v}}_{\text{1}\times \text{1}}(X)))$

6: **Dual-Branch Structure:**

7: $X_{\text{1}}\leftarrow Block_{\text{2}}(Block_{\text{1}}(X))$         ⊲ Deep branch

8: $X_{\text{2}}\leftarrow Block_{\text{3}}(X)$             ⊲ Shallow branch

9: $W\leftarrow \sigma (\text{Con}{\text{v}}_{\text{1}\times \text{1}}(X_{\text{1}}-X_{\text{2}}))$        ⊲ Difference

10: **Feature fusion and compression:**

11: ${\tilde{X}}_{\text{1}}\leftarrow X_{\text{1}}⊗(\text{1}+W)$          ⊲ Modulation

12: $X_{F}\leftarrow Block_{\text{4}}({\tilde{X}}_{\text{1}}⊗X_{\text{2}})$        ⊲ Integrate features

13: **Updating Feature map:**

14: $X_{GDC}\leftarrow \text{Con}{\text{v}}_{\text{1}\times \text{1}}(X)+X_{F}$       ⊲ Residual summation

15: **return** $X_{GDC}$


$$\begin{array}{c} X_{F}=Block_{\text{4}}({\tilde{X}}_{\text{1}}⊗X_{\text{2}}) \end{array}$$ (10)

$$\begin{array}{c} X_{\text{GDC}}=\text{Con}{\text{v}}_{\text{1}\times \text{1}}(X)⊕X_{F} \end{array}$$ (11)

where $\text{Bloc}{\text{k}}_{\text{4}}$ represents the composite feature extraction unit.

Algorithm 2 illustrates the overall operation of the GDC unit, where $\frac{\text{1}}{\text{3}}\sum_{d=\text{1}}^{\text{3}}\left(.\right)$ denotes the averaged fusion of convolution results under three different dilation rates (1, 2, 3), $\text{Bloc}{\text{k}}_{i}$ refers to a composite feature extraction block sequentially composed of a MDBConv unit, a GroupNorm layer (GN(·)), and a ReLU activation function.

### 3.4. EE-CSFF module

With deepening the backbone, spatial details of small objects could be easily lost due to repeated downsampling and feature map compression, which limits effective cross-scale feature interaction. The original YOLO11n’s neck employs a Path Aggregation Network (PAN) to fuse semantic and spatial features through fixed top-down upsampling and lateral connections. However, this fixed fusion path does not explicitly align features across layers, allowing deep semantics to dominate over shallow features. Consequently, the representation of small objects is weakened, leading to reduced detection performance.

**Fig 5:**
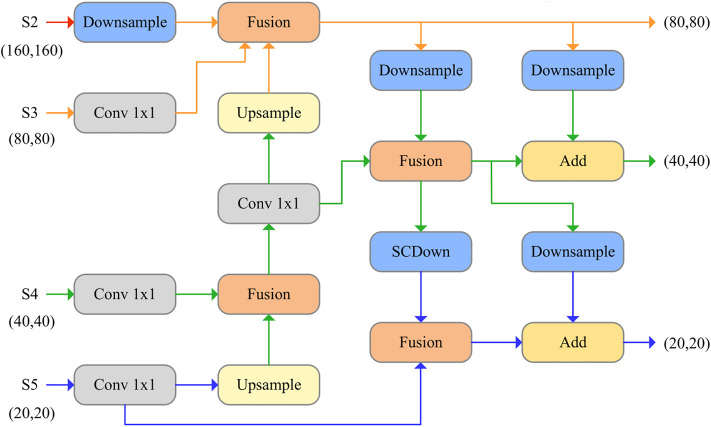
The detailed architecture of the newly designed EE-CSFF module, utilized by the proposed models. Backbone inputs (S2–S5) span spatial dimensions from (160, 160) to (20, 20) with strides 4, 8, 16, and 32. Overall, this architecture aggregates low-level spatial details with high-level semantics, preserving critical representations for small-lesion detection. Colored arrows (red, orange, green, blue) trace hierarchical flows. Grey blocks (Conv) align channels; yellow and blue blocks denote Upsample and Downsample operations, respectively. “Fusion” performs concatenation followed by GDCF, while “Add” signifies an element-wise addition. Outputs are generated at three scales: (80, 80), (40, 40), and (20, 20).

To address this, inspired by the CNN-based Cross-scale Feature Fusion (CCFF) module in RT-DETR [24], we redesigned the multi-scale fusion pyramid of YOLO11n and propose here the EE-CSFF module (Fig 5), which enhances cross-scale information interaction and preserves shallow features through channel alignment and multi-path fusion. This novel module structure allows to maintain top detection performance while reducing the model’s parameter count and computational overhead.

Specifically, EE-CSFF applies convolutions to features S3–S5 (c.f., Fig 5) to unify their channels to 256, reducing the computational cost of fusion and simplifying subsequent interactions. For the high-resolution S2 feature, downsampling is first applied before fusion in order to balance spatial detail preservation with scale alignment. Explicit channel alignment eliminates the need for additional up/downsampling during concatenation or addition, preserves critical shallow features, and streamlines cross-scale fusion.

In the top-down refinement path, fused features from shallower branches typically require downsampling to match the lower resolution of deeper features, such as S5. Direct downsampling, however, often increases computational cost and parameter count. To address this, we employed the lightweight SCDown [25] structure, which first reorganizes channel information via a convolution, followed by spatial downsampling through a depthwise separable convolution. This strategy reduces computational cost while preserving critical semantic information, enhancing deep-feature representation.

Furthermore, S3 (80, 80) and S4 (40, 40) features are downsampled and fused with corresponding S4 (40, 40) and S5 (20, 20) features, respectively, through an element-wise addition. This cross-layer residual fusion improves consistency between adjacent deep features, optimizes gradient flow, and reduces redundant computation. In summary, EE-CSFF alleviates the loss of shallow key information and deep feature mismatch through explicit channel alignment, lightweight design, and efficient cross-scale residual fusion. This approach significantly enhances the proposed models’ perception of small objects while maintaining overall model compactness.

### 3.5. LAMP method

To reduce redundant parameters while keeping the accuracy loss minimal and lowering computational, memory, and hardware demands, we adopted the LAMP method [21], a weight-magnitude-based, hierarchical pruning that does not require additional hyperparameters. For each layer, LAMP assigns a relative-importance score to each connection. This score is defined as the ratio of the connection’s squared magnitude to the cumulative squared magnitude of all remaining unpruned connections in the same layer. Under a global sparsity constraint, it ensures that each layer retains at least one highest-magnitude connection while accounting for inter-layer contribution differences. By incorporating a model-aware distortion metric based on the $l_{\text{2}}$ norm, LAMP minimizes pruning-induced output distortion and enables more effective accuracy recovery during retraining, while remaining efficient through standard tensor operations. The LAMP score is formulated as follows:


$$\begin{array}{c} \text{Score}\left(u;𝐖\right)=\frac{(W[u]{)}^{\text{2}}}{\sum_{v\geq u}(W[v]{)}^{\text{2}}} \end{array}$$ (12)

where W[u] denotes the weight at index u, and the denominator represents the sum of squares of all the remaining weights from index u to the end of the layer. The LAMP pruning decision is expressed mathematically as follows:


$$\begin{array}{c} (W[u]{)}^{\text{2}}>(W[v]{)}^{\text{2}}\Rightarrow \text{score}(u;𝐖)>\;\text{score}\left(v;𝐖\right) \end{array}$$ (13)

**Fig 6:**
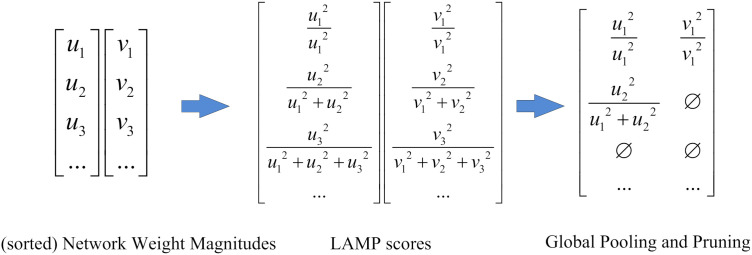
The computation workflow of LAMP scores and their application in global pruning. The process involves three sequential steps. First, network weights from different layers (denoted by variables *u* and *v*) are sorted by magnitude. Second, LAMP scores are calculated by normalizing each squared weight by the cumulative sum of squares of all surviving weights within the respective layer. Finally, global pooling evaluates these scores across all layers, whereby the weights falling below a unified threshold are pruned (represented by the null symbol, ∅). Overall, this analytical mechanism automatically determines optimal layer-wise sparsity, effectively removing redundant parameters without requiring manual threshold tuning.

As shown in (12), the logical relationship in (13) naturally holds. Specifically, when $(W[u]{)}^{\text{2}}>(W[v]{)}^{\text{2}}$, the LAMP score of W[u] is also higher than that of W[v]. This indicates that weights with larger magnitudes correspond to higher LAMP scores, while those with relatively lower scores are more likely to be pruned. The computation workflow of LAMP scores and their application in global pruning is illustrated in Fig 6.

### 3.6. Formal analysis of computational complexity

To conceptually justify the lightweight nature of the proposed DCL-YOLO model, we formalize the asymptotic complexity of the core operator, GDC, using O notation. Compared to standard convolutions with time complexity of $O(H\cdot W\cdot C_{in}\cdot C_{out}\cdot K^{\text{2}})$, GDC replaces computationally expensive dense feature extractions with MDBConv. By mathematically decoupling spatial aggregation from channel mixing into parallel multi-dilation depthwise convolutions (DWConv) and pointwise (1×1) projections, it structurally bounds the asymptotic time complexity to $O(H\cdot W\cdot C\cdot (K^{\text{2}}+C_{out}))$. Mathematically, the theoretical computational reduction ratio is:


$$\begin{array}{c} \text{Reduction}\;\text{Ratio}\approx \frac{\text{1}}{C_{out}}+\frac{\text{1}}{K^{\text{2}}} \end{array}$$ (14)

Given that $C_{out}\gg \text{1}$ and $K^{\text{2}}>\text{1}$ in typical architectures, this formal analysis validates a consistent reduction in theoretical GFLOPs upon incorporating this ultra-lightweight operator into the GDCF module.

## 4. Experiments

### 4.1. Datasets

#### 4.1.1. ALDSOD.

As a main dataset in this study, we utilized a constructed ALDSOD dataset for apple-leaf disease detection. ALDSOD is based on the AppleLeaf9 dataset [26] and supplemented with high-quality images from the Ai Studio dataset [27], comprising a total of 3,206 images covering four representative small-object diseases: Alternaria leaf spot, Rust, Gray spot, and Frogeye leaf spot.

**Fig 7:**
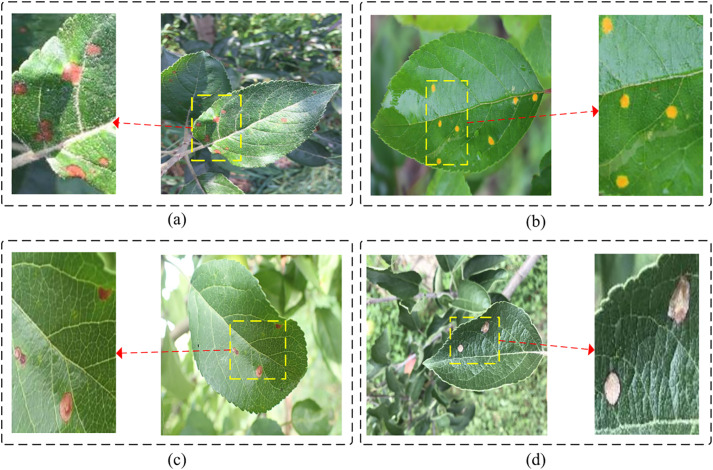
Representative sample images of the AppleLeaf9 dataset (CC BY 4.0) illustrating four apple-leaf disease categories. (a) Alternaria leaf spot, (b) Rust, (c) Frogeye leaf spot, and (d) Grey spot. The magnified crops highlight the minute scale, varied morphology, and subtle visual features of the diseases against complex natural backgrounds. Overall, these visual examples demonstrate the inherent challenges of small-object detection in real-world agricultural scenarios, justifying the need for the proposed architectural enhancements. In each panel, yellow dashed boxes indicate the specific regions of interest on the leaves, while red dashed arrows point to the corresponding magnified views of the lesions.

The AppleLeaf9 dataset integrates four publicly available sources: PVD [28], ATLDSD [29], PPCD2020 [3], and PPCD2021 [3], with approximately 94% of the images captured under real orchard conditions, covering diverse environmental scenarios, including sunny, cloudy, and post-rain weather, as well as various illumination angles, such as front lighting, back lighting, and side lighting. Representative sample AppleLeaf9 images, illustrating the four diseases, are shown in Fig 7.

To ensure strict annotation consistency, all original 3,206 images, included in the constructed ALDSOD dataset, were meticulously annotated with bounding boxes using LabelImg and saved in standard YOLO TXT format across the four disease categories. To rigorously prevent any potential data leakage, the ALDSOD dataset preparation followed a strict operational pipeline. Specifically, it was first partitioned into mutually exclusive training, validation, and test sets in a 7:1.5:1.5 ratio.

Given the relatively limited scale of the original datasets, utilized in constructing the ALDSOD dataset, and the highly complex nature of real orchard environments, offline data augmentation was subsequently applied independently to each respective set. Specifically, a stochastic compositional augmentation strategy was employed. Each image was sequentially evaluated against multiple candidate techniques with independent probabilities, allowing for random combinations of transformations on a single image. The candidate techniques included: brightness adjustment (with a randomized factor between 0.7 and 1.3), dynamic translation (spatially restricted to 33% of the bounding box margin to prevent target clipping), random rotation (within ± 5° coupled with a 0.7–0.8 scaling factor), random horizontal and vertical flipping, and Gaussian noise addition (with variance of 0.01). For the training set, this expanded sample diversity to prevent overfitting. For the validation and test sets, this deliberate augmentation introduced simulated environmental variations to ensure a comprehensive evaluation under diverse conditions, as detailed in Table 1. This strict decoupling strategy guarantees that augmented variants of any original image are strictly confined to their own set, ensuring zero overlaps between the training and test distributions. Following this pipeline, the ALDSOD’s size was effectively doubled to 6,412 images without any cross-contamination, guaranteeing that the disease lesions evaluated in the test set remain strictly unseen during training.

**Table 1:** Distribution of apple-leaf disease images in the constructed ALDSOD dataset before (‘orig.’) and after (‘augment.’) independent data augmentation performed.

Disease	Training set’s images (orig./ augment.)	Validation set’s images (orig./ augment.)	Testing set’s images (orig./ augment.)
Alternaria leaf spot	475/950	102/204	103/206
Rust	672/1344	144/288	145/290
Frogeye leaf spot	618/1236	132/264	133/266
Grey spot	477/954	102/204	103/206
***Total images***	***2242/4484***	***480/960***	***484/968***

**Fig 8:**
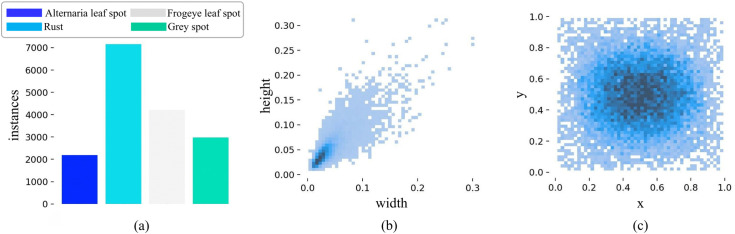
Statistical analysis of the constructed ALDSOD dataset. (a) A bar chart illustrating instance counts across four disease categories: Alternaria leaf spot (dark blue), Rust (cyan), Frogeye leaf spot (light grey), and Grey spot (green). The y-axis denotes total annotated instances. (b) A density scatter plot of lesion dimensions. The axes represent normalized bounding boxes’ width and height, with darker regions indicating higher frequency. (c) A spatial heatmap of normalized bounding boxes’ centers. Overall, the pronounced clustering near the origin in (b) and the dense central distribution in (c) quantitatively confirm that the ALDSOD dataset is heavily dominated by minute lesions, validating the need for specialized small-object detection models.

To gain a comprehensive understanding of the characteristics of the main dataset (ALDSOD) utilized in the experiments, a statistical analysis was performed, as illustrated in Fig 8. Specifically, it shows: (a) the distribution of the four disease categories, with Rust representing the largest proportion, followed by Frogeye leaf spot, Grey spot, and Alternaria leaf spot; (b) the distribution of normalized bounding boxes’ widths and heights, indicating that most instances are small in size, thereby highlighting the inherent challenges of small-object detection; and (c) the spatial heatmap of normalized bounding boxes centers, demonstrating that the majority of lesions are concentrated near the image center, while relatively few are located near the edges.

Overall, these statistical findings indicate that the ALDSOD dataset consists predominantly of small, centrally located lesions, confirming its suitability for research on small-object detection.

#### 4.1.2. GWHD.

The Global Wheat Head Detection (GWHD) dataset [30] was used for cross-domain validation of generalization capabilities of the proposed DCL-YOLO model.

This dataset is the results of a collaborative effort involving several global leaders in plant phenomics, including Arvalis, INRAE, RRes, and ETHZ in Europe, and USask in North America. By providing a vast collection of wheat head images from different continents, the dataset covers a wide range of environmental conditions and wheat varieties. Its multi-source nature ensures the generalization ability of the trained object detection models.

**Fig 9:**
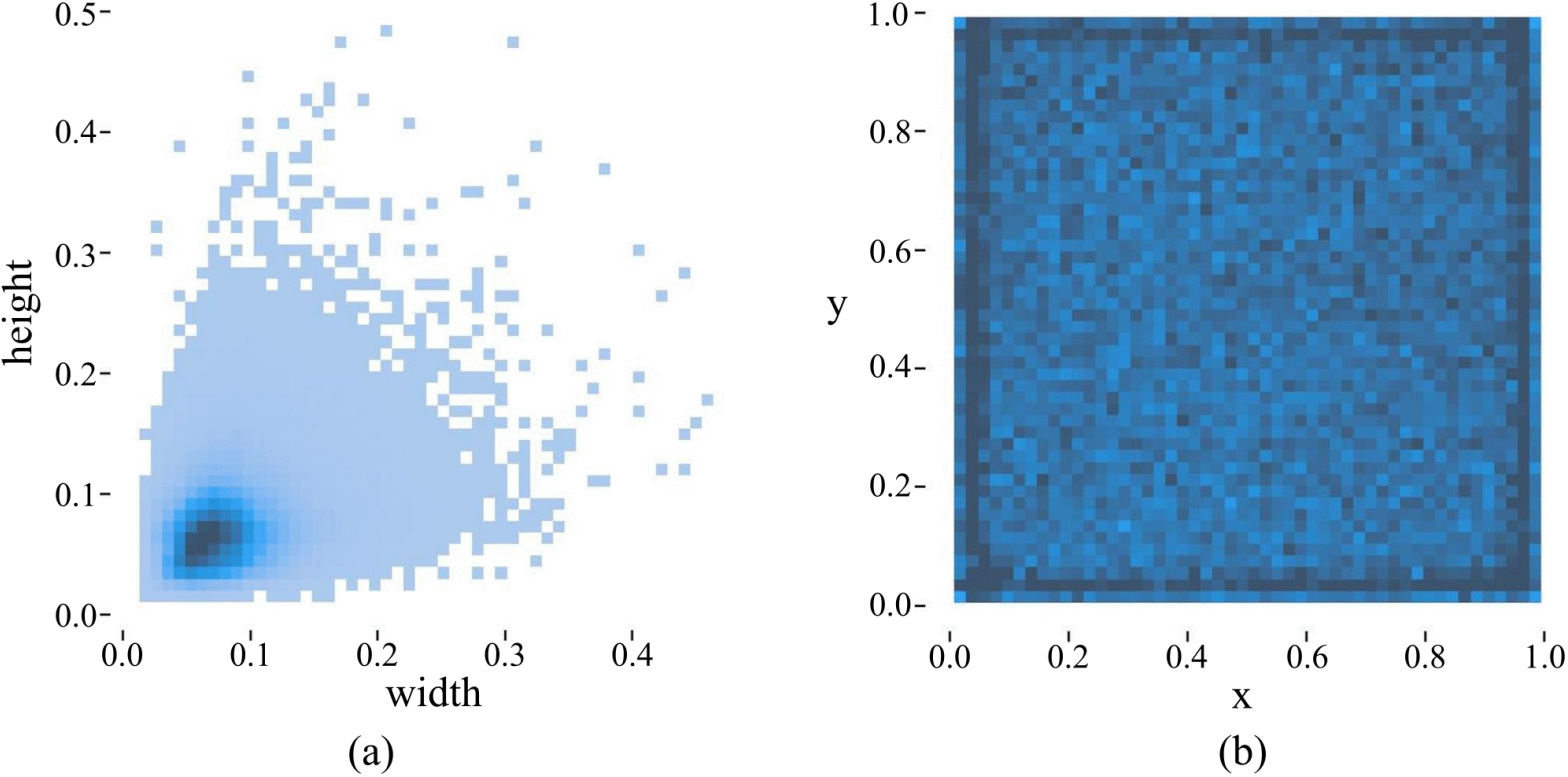
Bounding box statistics of the GWHD dataset. (a) A density scatter plot of lesion dimensions. The axes represent normalized bounding boxes’ width and height, with darker regions indicating higher frequency. (b) A spatial heatmap of normalized bounding boxes’ centers. As observed, targets are widely distributed without central concentration (panel b), while being of predominantly minute type with varying aspect ratios, appearing as irregular rectangles and squares (panel a). These geometric variations pose a strict challenge to model localization, ensuring a rigorous and spatially unbiased evaluation.

To directly address concerns regarding potential center-biased spatial priors, the conducted statistical analysis of the GWHD dataset (Fig 9) reveals that its targets are widely distributed across the image plane rather than being concentrated in the center.

### 4.2. Experimental environment and configuration

The experiments were conducted using the SGD optimizer with a batch size of 32 for 200 training epochs. The initial learning rate was set to 0.01, with a momentum of 0.937 and a weight decay of 0.0005, and a linear learning rate decay schedule preceded by a 3-epoch linear warmup. Input images were resized to 640×640 pixels. During training, comprehensive data augmentations, including Mosaic (specifically disabled in the final 10 epochs), random horizontal flipping, HSV (hue, saturation, value) variations, and random erasing were applied. An early stopping strategy was applied, such that model training was terminated if detection performance on the validation set did not improve for 15 consecutive epochs. To ensure experimental fairness, no pre-trained weights were used. Furthermore, the structural validity of the EE-CSFF neck and optimal configurations for parameterized components were determined through a systematic coarse-to-fine optimization process. Initial parameter ranges were informed by empirical heuristics, while the final values were established strictly through ablation studies (further detailed in Subsection 4.6) to maximize the efficiency−accuracy trade-off. Specifically, the optimal settings include a split ratio α=0.75 for DAFCM, a dilation sequence of (1, 2, 3) for MDBConv, and a LAMP-2.0 compression ratio (reducing computation by 50%) for pruning. All experiments were conducted on the same machine, with additional environmental details provided in Table 2.

**Table 2:** Experimental environment.

Component	Denomination
Operating system	Windows 10
Programming language	Python 3.10
CPU	Intel Core i5-12600KF
GPU	NVIDIA GeForce RTX 4070 Super
CUDA	11.8

### 4.3. Evaluation metrics

To compare the proposed models with state-of-the-art models, we employed a range of metrics, including precision, recall, average precision (AP) for each class, and mean average precisions (mAP) across all classes (mAP_50_ and mAP_50:95_). mAP_50_ is the mAP computed at a single IoU threshold of 0.50, while mAP_50:95_ is averaged over multiple IoU thresholds from 0.50 to 0.95 with a step size of 0.05. These evaluation metrics are calculated as follows:


$$\begin{array}{c} P=\frac{TP}{TP\;+FP} \end{array}$$ (15)

$$\begin{array}{c} R=\frac{TP}{TP\;+FN} \end{array}$$ (16)

$$\begin{array}{c} AP=\int_{\text{0}}^{\text{1}}P(R)dR \end{array}$$ (17)

$$\begin{array}{c} mAP=\frac{\text{1}}{N}\sum_{\;i=\text{1}}^{\;N}{AP}_{i} \end{array}$$ (18)

$$\begin{array}{c} {mAP}_{\text{5}\text{0}}=\frac{\text{1}}{N}\sum_{\;i=\text{1}}^{\;N}\overset{\left(i\right)}{\underset{\text{5}\text{0}}{AP}} \end{array}$$ (19)

$$\begin{array}{c} {mAP}_{\text{5}\text{0}:\text{9}\text{5}}=\frac{\text{1}}{\text{1}\text{0}}\left({mAP}_{\text{5}\text{0}}+{mAP}_{\text{5}\text{5}}+\ldots +{mAP}_{\text{9}\text{5}}\right) \end{array}$$ (20)

where TP, FP, and FN denote the numbers of true-positive, false-positive, and false-negative cases, respectively, N denotes the total number of classes, and i denotes the class index.

Additionally, the models’ lightweight characteristics were evaluated using the weight file size, parameter count, and computational cost (GFLOPs). In comparative experiments, the inference speed was measured in frames per second (FPS) with a batch size of 32.

These complementary sets of metrics allowed us to comprehensively evaluate the practical performance of the compared models.

### 4.4. Model performance

#### 4.4.1. Comparisons with state-of-the-art models.

**Fig 10:**
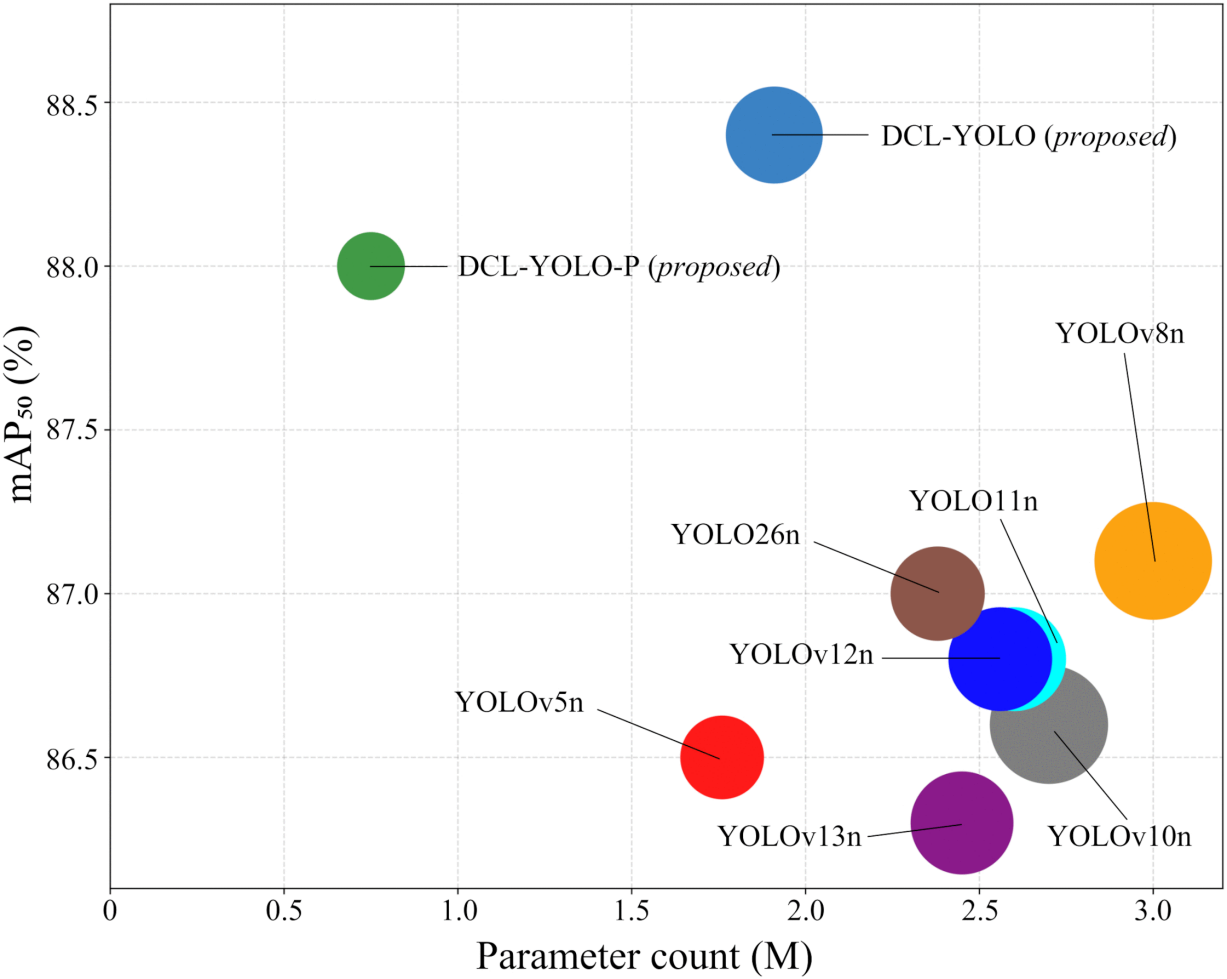
Accuracy–efficiency trade-off comparison. This bubble chart evaluates the proposed DCL-YOLO model and its pruned version (DCL-YOLO-P) against state-of-the-art lightweight YOLO versions, including the most recent one, i.e., YOLO26n. Heavier models (e.g., RT-DETR18 and YOLO11s) are excluded to maintain a strict focus on edge-deployable architectures. The x-axis presents the parameter count (in millions of parameters), whereas the y-axis indicates the mean average precision at an IoU threshold of 0.5 (mAP_50_). Colors differentiate the models, while the circle radii represent computational complexity in GFLOPs. Occupying the top-left quadrant, the proposed DCL-YOLO-P model achieves the highest accuracy-to-parameter ratio, demonstrating best balance for edge deployment.

In this subsection, we assess the performance of the proposed models on the ALDSOD dataset, in comparison to the state-of-the-art object detection models. The selection of models was driven by three motivations: (1) benchmarking the accuracy−efficiency trade-off against recent mainstream lightweight YOLO variants (YOLOv5n [31] YOLOv8n [32], YOLOv10n [25], YOLOv12n [33], YOLOv13n [34], YOLO26n [35], YOLO11n [17]); (2) evaluating architectural scalability by comparing the proposed nano-scale models with a heavier small-scale detector (YOLO11s); and (3) contrasting the computational efficiency of the CNN-based architecture of the proposed models against a modern Vision Transformer (RT-DETR-R18 [24]). The obtained results are displayed in Table 3 and Fig 10.

**Table 3:** Comparison results of the proposed DCL-YOLO model and its pruned version (DCL-YOLO-P) with state-of-the-art object detection models (based on a single representative run on the ALDSOD dataset).

Model	Precision (%)	Recall (%)	mAP_50_ (%)	mAP_50:95_ (%)	Parameter count (M)	GFLOPs	FPS (batch size = 32)
YOLOv5n	83.2	84.1	86.5	47.0	1.76	4.1	**1290.0**
YOLOv8n	84.4	81.2	87.1	46.8	3.00	8.1	929.4
YOLOv10n	**85.5**	79.8	86.6	47.1	2.70	8.2	725.7
YOLO11n	84.9	81.9	86.8	47.0	2.58	6.3	878.5
YOLO11s	85.2	81.1	87.3	47.0	9.41	21.3	381.9
YOLOv12n	83.0	82.5	86.8	47.4	2.56	6.3	577.3
YOLOv13n	84.9	80.1	86.3	46.6	2.45	6.2	389.2
YOLO26n	85.1	81.4	87.0	46.4	2.38	5.2	933.0
RT-DETR18	84.7	83.4	87.0	47.4	19.88	57.0	183.3
DCL-YOLO (*proposed*)	83.9	**84.6**	**88.4**	**47.8**	1.91	5.5	791.7
DCL-YOLO-P (*proposed*)	82.9	84.4	88.0	47.5	**0.75**	**2.7**	1012.3

As shown in Table 3, the proposed DCL-YOLO and DCL-YOLO-P models demonstrate superior performance across three (out of four) evaluation metrics, occupying first and second place, respectively. Specifically, DCL-YOLO (resp. DCL-YOLO-P) achieves a recall value of 84.6% (resp. 84.4%), an mAP_50_ value of 88.4% (resp. 88.0%), and an mAP_50:95_ value of 47.8% (resp. 47.5%), with only 1.91 M (resp. 0.75 M) parameters and 5.5 (resp. 2.7) GFLOPs, while maintaining high computational efficiency at 791.7 (resp. 1012.3) FPS. In the context of practical agricultural disease management, prioritizing recall is essentially critical to minimizing false negatives and preventing early-stage outbreaks of highly camouflaged minute lesions. The gain of DCL-YOLO (resp. DCL-YOLO-P) in recall, compared to mainstream YOLO models (YOLOv5n, YOLOv8n, YOLOv10n, YOLO11n, YOLOv12n, YOLOv13n, YOLO26n), equals to 0.5 (resp. 0.3), 3.4 (resp. 3.2), 4.8 (resp. 4.6), 2.7 (resp. 2.5), 2.1 (resp. 1.9), 4.5 (resp. 4.3), and 3.2 (resp. 3.0) percentage points, respectively. While these improvements in recall may seem numerically modest, they represent a favorable trade-off between detection capability and model compactness, fulfilling the strict SRAM constraints of embedded AI chips.

A detailed comparison with the baseline (YOLO11n) further highlights this viable balance. Although YOLOv11n slightly surpasses DCL-YOLO and DCL-YOLO-P in precision, the proposed models achieve a higher recall and consistently outperform it across mAP metrics. More importantly, DCL-YOLO-P (resp. DCL-YOLO) achieves this superior comprehensive performance while reducing the parameter count and GFLOPs respectively by 70.9% (resp. 26.0%) and 57.1% (resp. 12.7%).

Notably, compared to the most recently introduced YOLO version (YOLO26n), both proposed models (DCL-YOLO and DCL-YOLO-P) not only achieve substantial gains in recall (+3.2 and +3.0 percentage points, respectively), mAP_50_ (+1.4 and +1.0 percentage points, respectively), and mAP_50:95_ (+1.4 and +1.1 percentage points, respectively), but also require fewer parameters (19.7% and 68.5%, respectively), explicitly demonstrating their advanced architectural efficiency. Moreover, against the heavier RT-DETR18 (19.88 M parameters, 57 GFLOPs, 183.3 FPS), both proposed models exhibit substantially reduced model complexity, while achieving better overall detection performance (except for precision). While RT-DETR-R18 is undeniably powerful, its core mechanism—self-attention across large feature maps—inherently introduces quadratic computational complexity and substantial MAC. This translates to severe memory-bound latency bottlenecks on SRAM-constrained NPU devices. In contrast, the proposed models rigorously avoid these overheads, achieving a highly favorable trade-off between deployment feasibility and detection performance, compared to RT-DETR-R18.

To analyze scale differences, YOLO11s—a significantly larger and more powerful (small-scale) YOLO version—was also included in the performance comparison. Remarkably, both proposed models demonstrated highly comparable detection performance to YOLO11s (beating it on recall, mAP_50_, and mAP_50:95_), while utilizing just one-fifth and one-twelfth of its parameter count. This demonstrates that the proposed innovations effectively bridge the performance gap between nano- and small-scale models.

Furthermore, to comprehensively analyze architectural efficiency across different model scales, the heavier YOLO11s variant was introduced as a stronger modern baseline. Remarkably, DCL-YOLO achieves highly comparable detection accuracy to YOLO11s (88.4% vs.87.3% in mAP_50_) while consuming less than one-fourth of its parameter count (1.91 M vs. 9.41 M). This cross-scale comparison robustly proves that our architectural innovations effectively bridge the performance gap between nano and small scale models. Moreover, to address the performance of Transformer-based architectures, Table 3 includes RT-DETR-R18, a highly competitive real-time Vision Transformer. While RT-DETR-R18 is undeniably powerful, its core mechanism—self-attention across large feature maps—inherently introduces quadratic computational complexity and substantial MAC. This translates to severe memory-bound latency bottlenecks on SRAM-constrained embedded NPUs. In contrast, our highly optimized CNN-based DCL-YOLO strictly avoids these overheads, achieving a superior trade-off between deployment feasibility and detection accuracy (88.4% vs. 87.0%) with a fraction of the parameter capacity (1.91 M vs. 19.88 M).

After pruning, its DCL-YOLO-P version further reduces the parameter count to 0.75 M and GFLOPs to 2.7, with an increase of the inference speed to 1012.3 FPS, with only a marginal decrease of mAP_50_ to 88.0%. Overall, by integrating an efficient network design with pruning, DCL-YOLO-P effectively balances detection performance, model compactness, and inference speed, providing a practical solution for real-time apple-leaf small-object detection.

**Fig 11:**
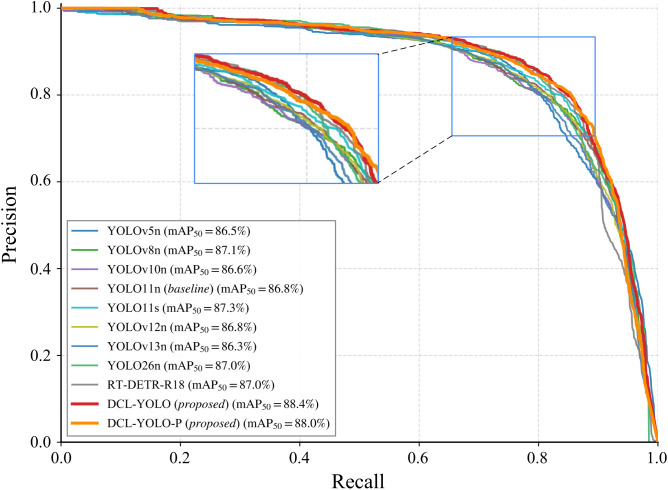
Precision–recall curves comparing the proposed models against state-of-the-art models. The x-axis and y-axis represent recall and precision, respectively. Distinct colored lines denote various YOLO architectures and RT-DETR-R18, with the legend detailing their mAP_50_ values. A blue inset box provides a magnified view of the upper-right region, clearly illustrating the performance gaps. By encompassing the largest area under the curve, the proposed DCL-YOLO and DCL-YOLO-P models achieve the highest mAP_50_, demonstrating superior detection robustness and accuracy.

The precision–recall curves, shown in Fig 11, demonstrate that DCL-YOLO and its pruned version, DCL-YOLO-P, consistently maintain stable detection capabilities comparable to the state-of-the-art models, validating the effectiveness of the proposed architectural enhancements even after pruning.

#### 4.4.2. Statistical significance and stability analysis.

To confirm that the performance gains of DCL-YOLO stem from architectural advancements rather than from random training variations, a rigorous statistical analysis was conducted. Specifically, both the baseline and the proposed models were re-evaluated over five independent runs using varying random initialization seeds. The quantitative results, including the mean and standard deviation (SD) for key metrics, are summarized in Table 4. As illustrated, the proposed models (DCL-YOLO and DCL-YOLO-P) consistently demonstrated superior performance over the baseline, according to all evaluation metrics. Notably, the error bounds (mean ± SD) of the proposed models and the baseline do not overlap for the core comprehensive metrics (mAP_50_ and mAP_50:95_), demonstrating consistent performance margins. The absence of overlapping variance intervals in these decisive metrics explicitly confirms that the performance gains—particularly in the localization-sensitive mAP_50:95_—are statistically significant and highly reproducible. This analysis demonstrates the structural robustness of the proposed models’ architecture in mitigating training stochasticity and ensuring precise detection in complex orchard environments.

**Table 4:** Statistical stability analysis (mean ± SD) of model performance over five independent runs on the ALDSOD dataset.

Model	Average precision (%)	Average recall (%)	Average mAP_50_ (%)	Average mAP_50:95_ (%)
YOLO11n (*baseline*)	84.3 ± 1.0	82.2 ± 0.7	86.9 ± 0.3	46.9 ± 0.4
DCL-YOLO (*proposed*)	**84.6 ± 1.7**	**83.8 ± 1.8**	**88.4 ± 0.2**	**47.7 ± 0.4**
DCL-YOLO-P (*proposed*)	82.7 ± 1.3	83.9 ± 1.3	87.9 ± 0.1	47.3 ± 0.3

#### 4.4.3. Visual study.

**Fig 12:**
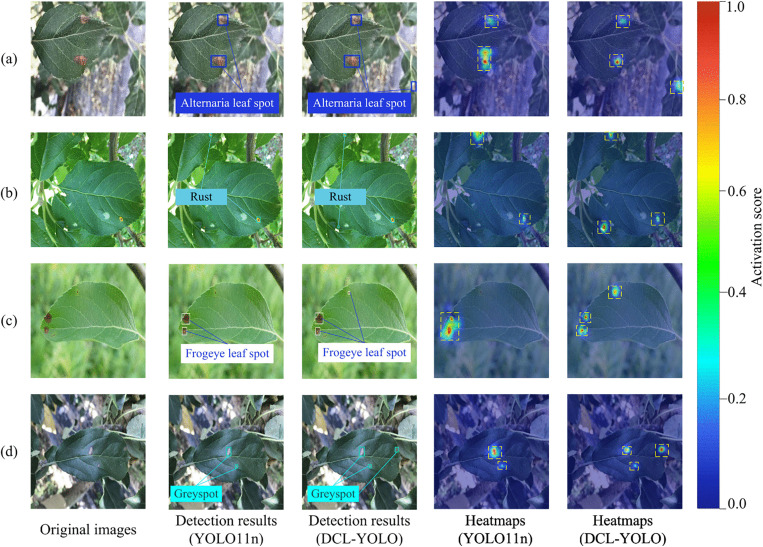
Qualitative performance comparison of the proposed DCL-YOLO model and the baseline (YOLO11n), performed on the ALDSOD dataset. Rows (a)–(d) display Alternaria leaf spot, Rust, Frogeye leaf spot, and Grey spot, respectively. Columns display original images, YOLO11n’s detection results, DCL-YOLO’s detection results, YOLO11n’s heatmaps, and DCL-YOLO’s heatmaps, respectively. The color bar (on the right) defines activation scores (0.0 to 1.0), where red indicates high model attention. Compared to the baseline, the proposed DCL-YOLO model demonstrates tighter bounding boxes and precisely concentrated activations on minute lesions. This visually confirms enhanced localization accuracy against complex backgrounds. (Images from AppleLeaf9, CC BY 4.0).

To better illustrate DCL-YOLO’s superior performance, Fig 12 presents its detection and heatmap results in comparison to the baseline (YOLO11n) for Alternaria leaf spot, Rust, Frogeye leaf spot, and Grey spot. DCL-YOLO’s heatmaps exhibit more focused activations around the lesion boundaries, indicating enhanced semantic understanding and suppression of background noise. In complex outdoor environments, DCL-YOLO can maintain stable and accurate highlighting of lesions, whereas the baseline often shows weak or misplaced activations outside of the lesion areas. In detection results, DCL-YOLO consistently identifies lesions with higher precision, accurately capturing shape and extent, while reducing false positives and missed detections, demonstrating superior detection performance and robustness under challenging conditions.

### 4.5. Cross-domain generalization analysis

To rigorously evaluate the generalization capabilities and architectural robustness of the proposed method beyond the specific domain of apple-leaf diseases, external validation was conducted on the GWHD dataset [30]. Although wheat heads differ morphologically from apple leaves, this dataset presents analogous agricultural visual challenges, such as high density, severe occlusion, and small-object scales.

Within this external validation framework, a strict cross-regional evaluation protocol was adopted to simulate severe environmental domain shifts. Specifically, models were trained exclusively on European data and evaluated on entirely unseen North American data, ensuring significant variances in lighting, crop varieties, and planting densities. Furthermore, for an impartial evaluation of structural efficiency, the unpruned model (DCL-YOLO) was compared against the baseline (YOLO11n). Quantitative results (Table 5) reveal that both proposed models outperform YOLO11n across all metrics. Notably, despite the challenging cross-domain setting, DCL-YOLO and DCL-YOLO-P achieve respectively a 3.7 and a 2.5 percentage-point improvement in recall, and a 2.2 and a 1.4 percentage-point increase in mAP_50_.

**Table 5:** Quantitative evaluation of model cross-domain generalization performance (based on a single representative run on the GWHD dataset).

Model	Precision (%)	Recall (%)	mAP_50_ (%)	mAP_50:95_ (%)
YOLO11n (*baseline*)	84.6	75.5	83.5	36.7
DCL-YOLO (*proposed*)	**84.9**	**79.2**	**85.7**	**38.6**
DCL-YOLO-P (*proposed*)	84.1	78.0	84.9	38.1

**Fig 13:**
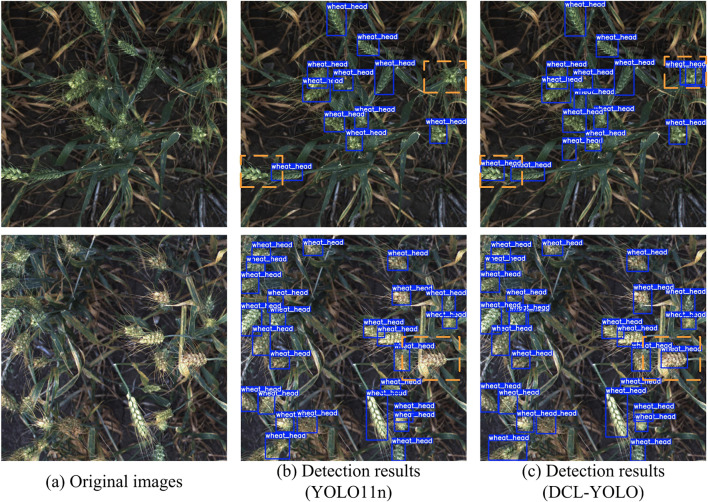
Qualitative performance comparison of the proposed DCL-YOLO model and the baseline (YOLO11n), performed on the GWHD dataset. Columns (a)–(c) display original images, YOLO11n’s detection results, and DCL-YOLO’s detection results, respectively. Solid blue boxes indicate detected wheat head bounding boxes. Orange dashed boxes specifically highlight regions where the baseline exhibits false negatives (missed detections), whereas the proposed DCL-YOLO model successfully localizes the heavily occluded targets. Overall, this visual evidence validates the improved recall and localization accuracy of DCL-YOLO when handling dense, overlapping small objects in complex agricultural environments.

Qualitatively, Fig 13 demonstrates that DCL-YOLO exhibits fewer missed detections in densely distributed regions. Furthermore, its ability to accurately localize minute objects across image peripheries (edges and corners) within the GWHD dataset indicates a successful learning of translation-invariant features, thus effectively alleviating concerns of center-bias reliance. Ultimately, this external validation provides strong evidence that the proposed architectural innovations avoid overfitting to specific orchard environments and exhibit stable generalization abilities across diverse agricultural scenarios.

### 4.6. Ablation study

To validate the effectiveness of the proposed model, we conducted ablation study experiments on the ALDSOD dataset to evaluate the contribution of each model component (individually or in combination with others). All experiments were performed under identical hardware conditions and training configurations, comprising nine experimental groups.

A summary of the results is provided in Table 6. The baseline model YOLO11n achieved 84.9% precision, 81.9% recall, 86.8% mAP_50_, and 47.0% mAP_50:95_, while requiring 6.3 GFLOPs and 2.58 M parameters.

**Table 6:** Ablation study experimental results, obtained by a single representative run on the ALDSOD dataset.

Model	DAFCM	GDCF	EE-CSFF	Pruning	Precision (%)	Recall (%)	mAP_50_ (%)	mAP_50:95_ (%)	GFLOPs	Parameter count (M)
YOLO11n (*baseline*)					84.9	81.9	86.8	47.0	6.3	2.58
	✔				82.3	84.3	87.6	47.1	6.4	2.62
		✔			84.7	82.0	87.4	46.6	6.2	2.51
			✔		84.5	82.2	86.9	47.2	5.5	1.88
	✔	✔			81.7	83.8	87.3	47.1	6.3	2.54
	✔		✔		**87.0**	81.1	88.0	47.5	5.5	1.92
		✔	✔		84.4	83.6	87.8	**47.9**	5.5	1.88
DCL-YOLO (*proposed*)	✔	✔	✔		83.9	**84.6**	**88.4**	47.8	5.5	1.91
DCL-YOLO-P (*proposed*)	✔	✔	✔	✔	82.9	84.4	88.0	47.5	**2.7**	**0.75**

In the first group of experiments, only individual module integration with the baseline was studied. Here, the DAFCM module component performed best according to recall and mAP_50_, while also exceeding the baseline performance on mAP_50:95_. The DAFCM module excels at preserving weak-response regions through an attention compensation strategy based on spatial and semantic flows. However, due to the introduction of a dual-path strategy in DAFCM, both GFLOPs and parameter count slightly increased, compared to the baseline.

The GDCF module component performed best in precision, reaching a slightly lower value than the baseline, while also exceeding its performance on recall and mAP_50_, with a minor decrease in GFLOPs and parameter count. These improvements come primarily from the GDCF’s ability to capture the response discrepancies between shallow and deep layers at corresponding spatial positions. By enhancing adaptive interactions across feature hierarchies, GDCF helps mitigate the loss of fine-grained information caused by weak shallow responses or excessive downsampling in deeper network layers.

Meanwhile, the EE-CSFF module enhanced cross-scale information flow and shallow feature retention by actively optimizing channel alignment and employing a multi-path fusion strategy. While exceeding the baseline performance on recall, mAP_50_, and mAP_50:95_ (the latter was also the best score among the three newly designed modules), it also significantly reduced computational cost w.r.t. GFLOPs (by 12.7%) and parameter count (by 27.1%).

Building on the above single-module evaluations, subsequently we investigated the performance of paired module combinations to assess potential synergies. When both DAFCM and EE-CSFF components were integrated, the resulting model achieved top so-far performance on precision, while also exceeding the baseline performance on mAP_50_ and mAP_50:95_.

The ‘DAFCM + EE-CSFF’ improvements, however, were accompanied by a slight drop in recall, compared to the baseline. Such a phenomenon is more likely attributed to the enhanced discriminative capability of the fused features, which reduces false positives by enforcing stricter decision boundaries, but at the same time may overlook certain small or weakly activated targets, thereby slightly compromising recall.

The ‘GDCF + EE-CSFF’ combination achieved the best so-far result in mAP_50:95_, while also exceeding the baseline performance on recall and mAP_50_, and maintained reductions of 12.7% in GFLOPs and 27.1% in parameter count, compared to the baseline.

The ‘DAFCM + GDCF’ combination achieved the best result in recall in this group of experiments, exceeding the baseline performance not only on recall but also on mAP_50_ and mAP_50:95_.

These results demonstrate that all three newly designed modules are capable of achieving a balanced trade-off between detection performance and lightweight model design when used in different pairwise configurations.

With the integration of all three novel modules, the final model (DCL-YOLO) and its pruned version (DCL-YOLO-P) maintained a lightweight profile with only 5.5 and 2.7 GFLOPs and 1.91 M and 0.75 M parameters, respectively, while reaching first and second place in recall and mAP_50_, and also outperforming the baseline in mAP_50:95_. Furthermore, after applying the proposed pruning strategy, the DCL-YOLO-P’s computational cost was further reduced by 50.9% and its parameter count dropped by 60.7%, compared to the full model (DCL-YOLO). Despite this significant compression, the pruned model (DCL-YOLO-P) still surpasses the baseline on three (out of four) evaluation metrics, demonstrating that the pruning strategy effectively minimizes performance loss while substantially lowering computational and storage overhead.

Beyond the macro-level module combinations discussed above, the internal configurations of each module—such as the channel split ratio in DAFCM, the gating parameters in GDCF, and the explicit fusion strategy in EE-CSFF—require meticulous optimization to fully justify our design choices. Detailed theoretical and empirical analyses of these specific settings are presented in subsequent subsections (4.6.1 to 4.6.3).

Furthermore, in the context of mainstream neck designs, the choice of fusion strategy is critical. Within the proposed EE-CSFF module, concatenation (‘Concat’) was deliberately selected over element-wise addition (‘Add’). From a theoretical perspective, directly applying an element-wise addition to merge upsampled deep features with shallow features forcibly mixes highly abstract semantics with fine-grained spatial details in the same latent space. For highly camouflaged minute lesions, this direct numerical accumulation causes severe semantic aliasing and blurs crucial high-frequency spatial boundaries. Conversely, the concatenation strategy strictly preserves these distinct multi-scale representations in independent channels, allowing the subsequent GDCF module to dynamically aggregate features without irreversible information corruption.

In summary, the proposed improvements allowed to outperform the baseline in both detection performance and model efficiency. Moreover, each newly designed module demonstrated a favorable balance between detection performance and design complexity under various integration configurations, validating the overall flexibility and effectiveness of design.

#### 4.6.1. Effects of split ratio in DAFCM.

Table 7 illustrates the influence of split ratio α, which controls the allocation between semantic features and spatial details, on the experimental results. The best performance of the proposed DCL-YOLO model was achieved when all values were set to 0.75. Conceptually, in this case, the majority of channels (75%) acquired sufficient semantic information to support the classification of micro-scale disease spots, while the remaining 25% of auxiliary channels retained essential spatial details to spatially anchor the abstract semantics. This configuration effectively prevented the loss of positional information during downsampling and channel compression. Such a balance enabled attention mechanisms to better highlight micro-scale lesions and contributed to optimal performance in both mAP_50_ and mAP_50:95_. Furthermore, the performance peak at this ratio marked a rigorous convergence point. Any deviation disrupted the critical balance between semantic capacity and spatial anchors, confirming the empirical upper bound was reached and rendering further tuning redundant.

**Table 7:** Effect of using different split ratios (α) in DAFCM on the detection performance of the proposed DCL-YOLO model (based on a single representative run on the ALDSOD dataset).

Split Ratio (α)	mAP_50_ (%)	mAP_50:95_ (%)	GFLOPs	Parameter count (M)
(0.25, 0.25, 0.25, 0.25)	87.9	47.4	**5.3**	**1.81**
(0.50, 0.50, 0.50, 0.50)	87.3	47.4	5.4	1.85
(0.75, 0.75, 0.75, 0.75) *chosen*	**88.4**	**47.8**	5.5	1.91
(0.25, 0.25, 0.75, 0.75)	87.9	47.4	5.5	1.90
(0.75, 0.75, 0.25, 0.25)	88.1	47.3	5.4	1.81

#### 4.6.2. Effects of dilation configurations in MDBConv.

As shown in Table 8, the dilation configuration (1, 2, 3) achieved the best overall balance, reaching the highest recall (84.6%), mAP_50_ (88.4%), and mAP_50:95_ (47.8%), while the setting (3, 5, 7) scored highest only in precision (85.4%). Conceptually, establishing an appropriate receptive field requires a delicate balance. The baseline configuration of (1, 1, 1) suffers from a restricted receptive field, struggling to capture sufficient contextual surroundings. Conversely, this suggests that using overly high dilation rates inherently induces a “gridding effect”—a sparse sampling phenomenon—, which may lead to loss of fine-grained lesion details by skipping continuous essential pixels, thereby weakening the model detection capability and causing a detrimental drop in recall for small apple-leaf spots.

**Table 8:** Effect of varying dilation rates on the detection performance of the proposed DCL-YOLO model (based on a single representative run on the ALDSOD dataset).

Dilation configuration	Precision (%)	Recall (%)	mAP_50_ (%)	mAP_50:95_ (%)
(1, 1, 1)	83.2	83.6	87.8	47.5
(1, 2, 3) *chosen*	83.9	**84.6**	**88.4**	**47.8**
(1, 3, 5)	83.6	84.1	88.0	47.8
(3, 5, 7)	**85.4**	82.2	87.8	47.6

Based on these results, the MDBConv units within each GDCF module were configured with the (1, 2, 3) dilation setting to ensure best balance between precision and recall for detection of apple-leaf disease spots.

#### 4.6.3. Evaluation of EE-CSFF against mainstream neck designs.

**Fig 14:**
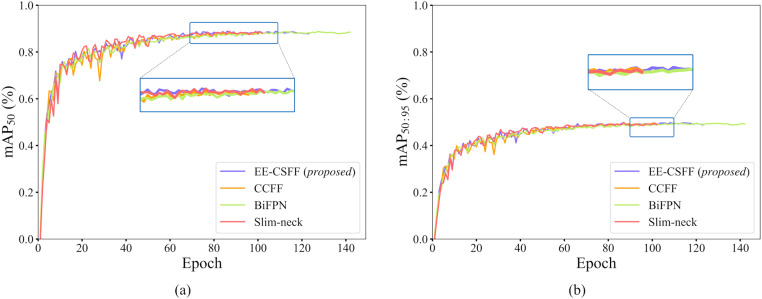
Regression performance and convergence speed comparison of the proposed EE-CSFF module against classical neck modules. Panels (a) and (b) track respectively mAP_50_ and mAP_50:95_ across training epochs. Distinct colored lines represent the compared modules (EE-CSFF, CCFF, BiFPN, Slim-neck). Blue inset boxes provide magnified views of the late-stage training phases to clarify marginal performance gaps. By consistently maintaining the highest trajectory in the final epochs, the proposed EE-CSFF module demonstrates superior training stability and peak accuracy, validating its effectiveness in multi-scale feature aggregation.

To evaluate the effectiveness of EE-CSFF, Fig 14 illustrates the regression performance and convergence speed throughout the training process of the newly designed EE-CSFF module compared to classical neck modules. These results demonstrate that EE-CSFF attains high accuracy while converging efficiently. The corresponding quantitative results are summarized in Table 9, showing that EE-CSFF achieves best results on mAP_50_ and mAP_50:95_, and second best results on GFLOPs and parameter count.

**Table 9:** Effectiveness of different neck modules on DCL-YOLO’s performance (based on a single representative run on the ALDSOD dataset).

Neck module	mAP_50_ (%)	mAP_50:95_ (%)	GFLOPs	Parameter count (M)
EE-CSFF (*proposed*)	**88.4**	**47.8**	5.5	1.91
CCFF [24]	87.6	47.7	**5.3**	**1.86**
BiFPN [36]	87.8	47.5	6.7	2.57
Slim-Neck [37]	87.2	47.4	6.4	2.77

Specifically, compared to CCFF [24], EE-CSFF improves mAP_50_ and mAP_50:95_ by 0.8 and 0.1 percentage points, respectively, with only a modest increase of 3.8% in GFLOPs and 2.7% in parameter count. Compared to BiFPN [36], EE-CSFF improves mAP_50_ and mAP_50:95_ by 0.6 and 0.3 percentage points, while also reducing GFLOPs by 17.9% and parameter count by 25.7%. Moreover, EE-CSFF increases mAP_50_ and mAP_50:95_ by 1.2 and 0.4 percentage points, respectively, compared to Slim-Neck [37], while reducing GFLOPs by 14.1% and parameter count by 31.0%.

These results clearly demonstrate that the newly designed EE-CSFF module, utilized by the proposed DCL-YOLO model, achieves optimal balance between accuracy and efficiency, establishing it as an effective and lightweight neck architecture for apple-leaf disease-spot detection.

#### 4.6.4. Comparative pruning experiments.

As shown in Table 10, different pruning strategies lead to varying degrees of performance degradation of the proposed DCL-YOLO-P model. There, “Speed-up level” refers to the computational compression ratio achieved under the LAMP pruning scoring algorithm. Specifically, LAMP‑2.0 means that the computation before pruning is twice that after pruning, and other configurations follow the same principle. The pruning process aims to eliminate structural redundancy—such as filters over-indexing on background noise—without crossing the network’s critical information bottleneck. As observed, the LAMP-2.0 configuration achieves an optimal trade-off point: it actually peaks mAP_50_ at 88.0% by acting as an implicit regularizer that effectively strips away redundant weights, while simultaneously slashing GFLOPs (to 2.7) and parameter count (to 0.75 M). However, pushing the speed-up beyond this point (e.g., LAMP-2.5 or 3.0) forcibly breaches the information bottleneck, causing the algorithm to aggressively prune essential feature representations for micro-lesions, leading to performance degradation. After evaluating multiple Speed-up configurations, LAMP-2.0 was selected as the final lightweight pruning setup, confirming it as the maximum tolerable compression boundary before structural capacity collapse.

**Table 10:** Performance of the proposed DCL-YOLO-P model under different speed-up levels (based on a single representative run on the ALDSOD dataset).

Speed-up level	mAP_50_ (%)	mAP_50:95_ (%)	GFLOPs	Parameter count (M)	Size (MB)
**Lamp**-1.5	87.9	**47.8**	3.7	1.00	2.43
**Lamp**-2.0 (*chosen*)	**88.0**	47.5	2.7	0.75	1.95
**Lamp**-2.5	87.3	47.2	2.2	0.62	1.69
**Lamp**-3.0	85.5	45.8	1.8	0.53	1.53
**Lamp**-3.5	86.1	45.6	**1.6**	**0.47**	**1.40**

## 5. Lightweight K230 embedded deployment

### 5.1. Deployment pipeline

Before deployment, several preparatory steps were required to establish a suitable runtime environment. First, CanMV IDE K230 (v4.0.7) was installed on the host machine to support firmware flashing and board debugging. Additionally, the nncase KPU runtime library (v2.9.0) [38] was configured to enable the subsequent*.kmodel* format conversion. Once the software environment was ready, the Rufus tool was used to write the firmware image (v1.3.0) to the K230 onboard storage via a TF card. Finally, the compiled *model.kmodel* file was transferred to the device, completing the essential setup for model inference.

**Fig 15:**
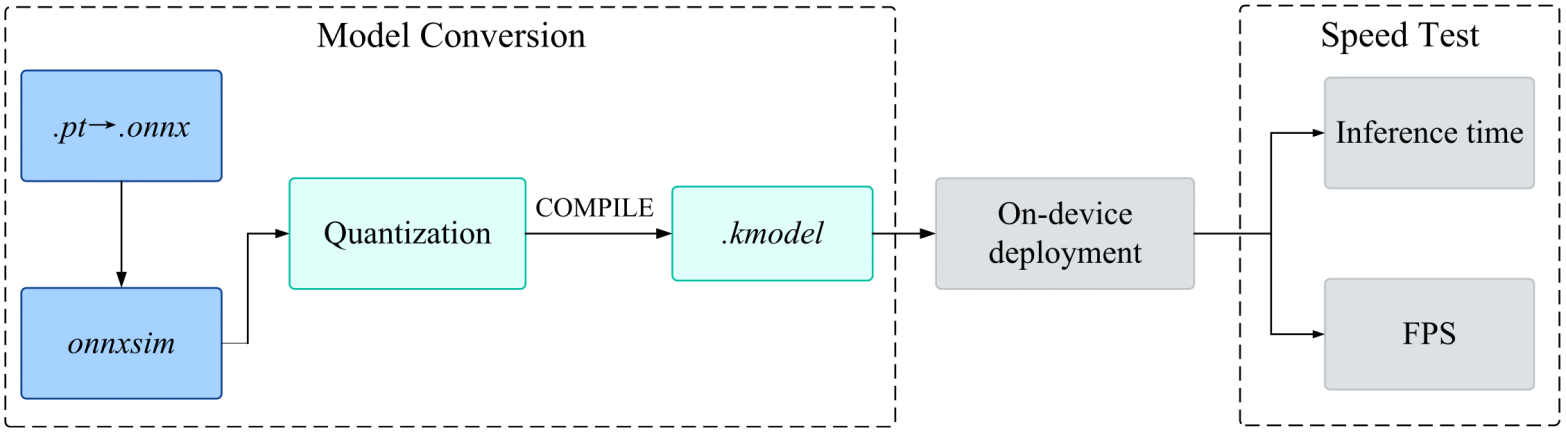
The pipeline of model deployment on the K230 edge platform. Dashed boundaries define logical processing stages. In the “Model Conversion” stage (left), PyTorch models (*.pt*) were exported via *onnxsim* and compiled into a quantized*.kmodel* format. Arrows indicate the sequential execution flow. The optimized model then underwent on-device deployment. Ultimately, the “Speed Test” stage (right) evaluates operational performance based on inference time and frames per second (FPS). Overall, this pipeline demonstrates the efficient translation of trained models for resource-constrained edge applications.


Following the completion of the environment setup, we proceeded to deploy the pruned DCL-YOLO version (DCL-YOLO-P) onto the CanMV K230 AI chip – a representative embedded device, aiming to enable real-time object detection in resource-constrained scenarios. As illustrated in Fig 15, the deployment pipeline consisted of three key stages − model conversion, on-device deployment, and speed test − all essential in ensuring reliable model operation within the hardware constraints of the K230 platform.

$$\begin{array}{c} Q=\text{round}\left(\frac{X}{S}+Z\right) \end{array}$$ (21)

where X denotes the original floating-point tensor, Q denotes the corresponding quantized integer tensor, and S and Z denote the scaling factor and zero-point offset, respectively.

The scale and zero-point parameters in the above formula can be computed as follows:


$$\begin{array}{c} s=\frac{x_{\text{max}}-x_{\text{min}}}{{\text{2}}^{\text{8}}-\text{1}} \end{array}$$ (22)

$$\begin{array}{c} Z=\text{round}\left(-\frac{x_{\text{min}}}{S}\right) \end{array}$$ (23)

where $x_{min}$ and $x_{max}$ denote the minimum and maximum values, respectively, of the mapped interval in the floating-point tensor X, and ${\text{2}}^{\text{8}}-\text{1}$ specifies the integer range supported by 8-bit quantization.

The validity of the above formulation relies on the appropriate selection of the threshold range $\left[x_{min},\;x_{max}\right]$. To achieve optimal mapping precision, we adopted a quantization strategy based on Kullback–Leibler (KL) divergence [39] to perform an optimized search over this interval. Specifically, the KL divergence-based method constructs the original (floating-point) activation distribution P and its approximate counterpart Q, obtained after quantization and dequantization, and minimizes the divergence between the two, as follows:


$$\begin{array}{c} D_{\text{KL}}(P∥Q)=\sum_{i}P(i)log\frac{P(i)}{Q(i)} \end{array}$$ (24)

where P(i) and Q(i) denote the probabilities of the i-th bin in the original distribution P and the approximate distribution Q, respectively. Smaller values of $D_{KL}$ indicate a more accurate approximation of P by Q.

By iterating over different clipping ranges $\left[x_{min},\;x_{max}\right]$ and computing the corresponding $D_{\text{KL}}(P∥Q)$, the clipping interval that minimizes the KL divergence can be identified. Compared with traditional Min–Max-based quantization, this approach more effectively preserves the primary characteristics of the tensor distribution.

Finally, the quantized*.onnx* model was compiled into the K230-specific*.kmodel* format and deployed to the target device. Real-time performance was validated through measurements of video frame rate and inference latency.

### 5.2. Comparative quantization experiments

Table 11 compares the effects of different quantization configurations on the DCL-YOLO-P’s size and inference performance during real-time video detection on a K230 development board. To circumvent memory-bound bottlenecks caused by the K230’s limited on-chip SRAM, the input resolution for deployment was optimized to 224 × 224 pixels to ensure real-time throughput. In Table 11, “Quantization scheme” denotes the quantization type of the model input and intermediate activations, “Weight” denotes the quantization type of weights, and “KLD” indicates whether the Kullback–Leibler Divergence (KLD) strategy was applied for threshold selection. All FPS values were measured under continuous video streaming conditions, while the detection time was evaluated using single-frame inputs.

**Table 11:** Performance comparison of different quantization schemes, applied to the proposed DCL-YOLO-P model.

KLD	Quantization scheme	Weight	Model size (MB)	FPS	KPU inference time (msec)
✔ *(chosen)*	Uint8	Uint8	1.24	**15.2**	**139**
	Uint8	Uint8	1.24	14.9	141
✔	Int8	Int8	**1.22**	14.3	141
	Int8	Int8	**1.22**	14.7	140
✔	Uint8	Int8	**1.22**	14.7	144
	Uint8	Int8	**1.22**	14.5	142
✔	Int8	Uint8	1.24	14.5	141
	Int8	Uint8	1.24	14.7	140

The obtained results demonstrate that the KLD-enabled Uint8/Uint8 configuration offers an optimal trade-off between compact model size (1.24 MB) and real-time processing speed (15.2 FPS). In comparison, although the Int8/Int8 configuration slightly reduces the model size (1.22 MB), its frame rate is noticeably lower than that of the Uint8/Uint8 configuration. Therefore, we ultimately adopted the KLD-enabled Uint8/Uint8 scheme as the preferred configuration for deployment, ensuring both model compactness and real-time detection performance.

### 5.3. Systematic Evaluation of Edge Deployment

**Fig 16:**
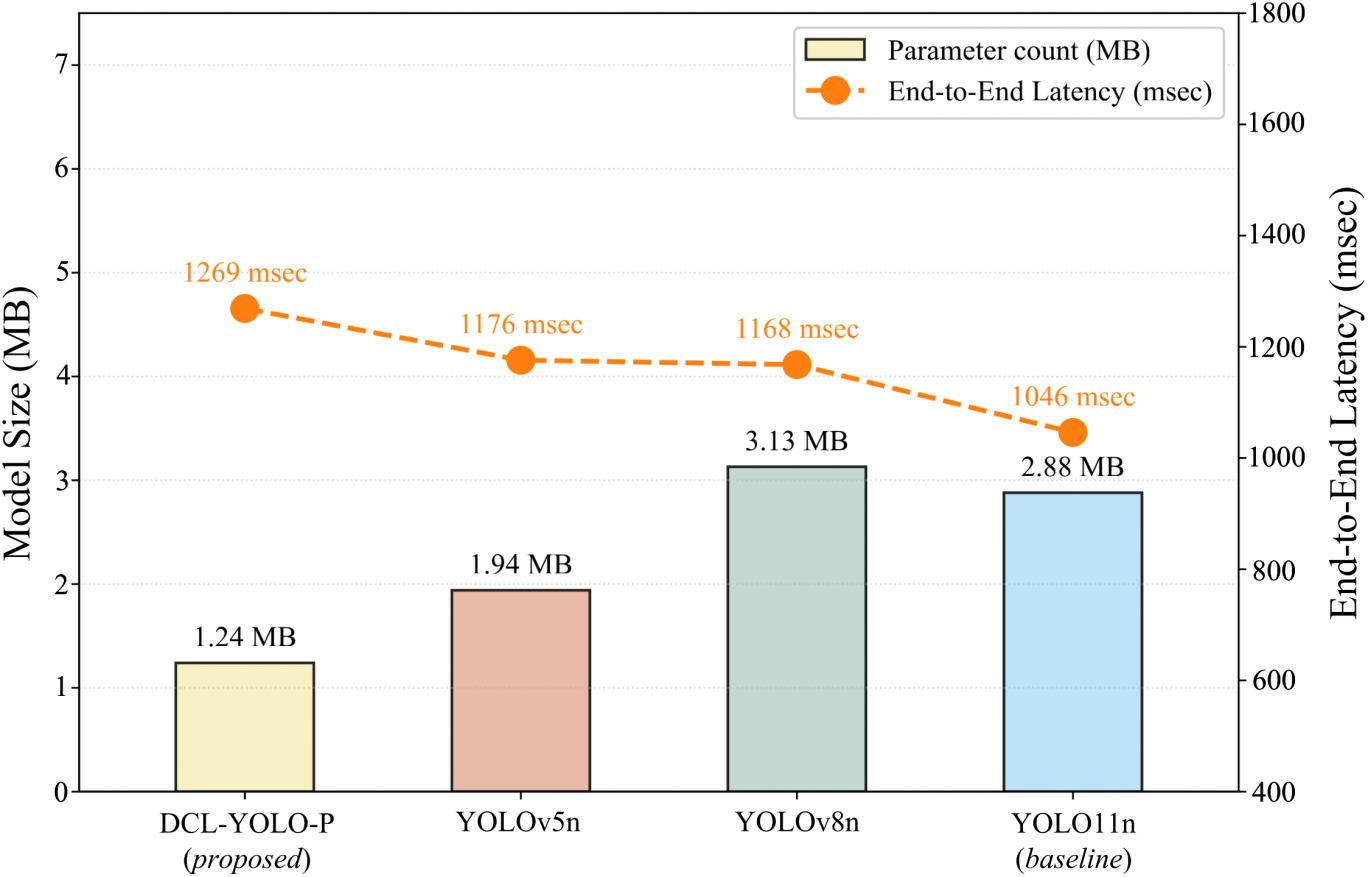
Performance trade-off between physical model size and end-to-end latency w.r.t. **DCL-YOLO-P evaluated against lightweight state-of-the-art models on the K230 edge device.** A dual-axis view is utilized: colored bars correspond to the left y-axis, indicating the model size in megabytes (MB), while the orange dashed line (mapped to the right y-axis) tracks latency in milliseconds. Although exhibiting a marginally higher latency, DCL-YOLO-P achieves a significantly reduced memory footprint (1.24 MB), demonstrating an exceptional memory−latency balance, proving its feasibility for deployment on highly resource-constrained edge hardware.

To systematically evaluate practical model deployment, the pruned proposed model (DCL-YOLO-P) was assessed against lightweight state-of-the-art models on the same K230 edge hardware (Table 12 and Fig 16). Notably, while FPS in Subsection 4.4.1 reflects theoretical GPU throughput via parallel batch processing (with a batch size of 32), the current subsection evaluates practical on-device latency under sequential, single-stream constraints (with a batch size of 1). The state-of-the-art models (YOLOv5n, YOLOv8n, YOLO11n) were selected for comparison with DCL-YOLO-P as they represent the most widely deployed lightweight models in industrial edge vision applications. Fig 16 visually illustrates the trade-off between physical model size and end-to-end latency. Regarding physical memory constraints, DCL-YOLO-P achieved an ultra-lightweight footprint of 1.24 MB, representing a 36.1%, 60.4% and 56.9% reduction compared to YOLOv5n, YOLOv8n, and YOLO11n, respectively. The highly compact footprint of DCL-YOLO-P offers a practical advantage for storage-restricted micro-edge nodes where on-chip memory is at a premium.

**Table 12:** Systematic comparison of deployment-oriented physical metrics on the K230 hardware platform.

Model	Model size (MB)	KPU inference time (msec)	End-to-end latency (msec)	Heap fluctuation (kB)
YOLOv5n	1.94	28	1176	2.22
YOLOv8n	3.13	**26**	1168	2.20
YOLO11n (*baseline*)	2.88	37	**1046**	**2.03**
DCL-YOLO-P (*proposed*)	**1.24**	139	1269	2.22

In terms of temporal performance, the compared state-of-the-art models exhibit faster inference times (~30 msec) primarily due to the specialized library-level optimizations and extensive graph fusion support provided by the K230 compiler (nncase) for standard YOLO architectures. In contrast, the novel architectural design of DCL-YOLO-P triggers an “operator fallback” to the CPU during compilation, as it has not yet benefited from such hardware-specific library accelerations, resulting in an inference time of 139 msec. Importantly, a detailed latency breakdown reveals that the remaining time within the total end-to-end latency of approximately 1.2 sec is predominantly consumed by unoptimized I/O operations and CPU-bound pre/post-processing, rather than the core network execution. Nevertheless, this total processing cycle is entirely sufficient for periodic agricultural monitoring tasks. Despite these trade-offs, the dynamic heap fluctuation for all models remains strictly bounded at an extremely low level (2.03–2.22 kB). This confirms the high scheduling stability of the deployment pipeline and ensures a negligible memory leak risk within the hardware ceiling.

## 6. Conclusion

In this article, an in-depth analysis of the characteristics of subtle lesions on apple leaves has been conducted and lightweight small-object detection models have been proposed, namely DCL-YOLO and its pruned version (DCL-YOLO-P). The proposed models were systematically optimized in three stages: feature extraction, feature enhancement, and feature fusion. In the feature extraction stage, newly designed DAFCM modules are used to adaptively compensate for the loss of semantic information in deep layers and spatial information in shallow layers, through a dual-path strategy, thereby enhancing the models’ feature matching capability for small objects. In the feature enhancement stage, novel GDCF modules are utilized to dynamically strengthen the representation of small objects and weak-response regions using a differential-aware gating mechanism. In the feature fusion stage, a newly designed EE-CSFF module is used to replace the original neck structure of the baseline (YOLO11n). This module explicitly aligns feature channels and employs multi-path fusion to enhance cross-scale information flow and preserve shallow features, while effectively reducing computational complexity.

On the constructed ALDSOD dataset, the proposed DCL-YOLO and DCL-YOLO-P models demonstrated superior performance over state-of-the-art object detection models. Compared to the baseline, they improved the values of three (out of four) evaluation metrics, while also being able to reduce the parameter count by 26.0% and 70.9%, and GFLOPs by 12.7% and 57.1%, respectively. Compared with mainstream lightweight detectors, both proposed models demonstrated superior detection performance w.r.t. small objects, while also introducing a smaller computational overhead.

The pruned model version (DCL-YOLO-P) was successfully deployed on an embedded platform, where it occupied only 1.24 MB and achieved a video inference speed of 15.2 FPS, demonstrating its feasibility and practicality for real-time edge inference.

Moreover, to address potential concerns regarding model robustness and cross-domain generalization, external validation was conducted on the Global Wheat Head Detection (GWHD) dataset using a strict cross-regional evaluation protocol. Both proposed models successfully outperformed the baseline (YOLO11n) across all evaluated metrics (except for precision achieved by the pruned DCL-YOLO-P model) on unseen target domains, proving that the proposed structural innovations are not overfitted to a specific dataset but possess stable generalization capabilities.

The proposed pruned DCL-YOLO-P model is well-suited for integration into automated orchard monitoring systems, such as Unmanned Aerial Vehicles (UAVs) and mobile handheld devices. By providing real-time, on-device disease identification, it enables precision agricultural practices by facilitating localized treatment and reducing manual labor. Despite these achievements, fully suitable public datasets with precise bounding-box annotations for subtle apple-leaf diseases are still relatively scarce, limiting extensive intra-species validation. Therefore, a cautious approach is required regarding immediate large-scale deployment across highly diverse real-world orchards. Our future work will focus on expanding the constructed dataset with more diverse in-field images and exploring multi-modal fusion strategies that integrate visual data with environmental sensors (e.g., temperature and humidity) to further verify and improve practical applicability. Additionally, exploring hardware-aware, ultra-lightweight Vision Transformers tailored for edge agricultural devices remains a highly promising direction to further push the boundaries of deployment efficiency.
